# How osteogenic is dexamethasone?—effect of the corticosteroid on the osteogenesis, extracellular matrix, and secretion of osteoclastogenic factors of jaw periosteum-derived mesenchymal stem/stromal cells

**DOI:** 10.3389/fcell.2022.953516

**Published:** 2022-10-31

**Authors:** Felix Umrath, Achim Pfeifer, Wanjing Cen, Marina Danalache, Siegmar Reinert, Dorothea Alexander, Andreas Naros

**Affiliations:** ^1^ Clinic for Oral and Maxillofacial Surgery, University Hospital Tübingen, Tübingen, Germany; ^2^ Clinic for Orthopaedic Surgery, University Hospital Tübingen, Tübingen, Germany

**Keywords:** dexamethasone, osteogenic differentiation, mesenchymal stem/stromal cells, periosteum, osteoclast, extracellular matrix

## Abstract

Dexamethasone (dexa) is commonly used to stimulate osteogenic differentiation of mesenchymal stem/stromal cells (MSCs) *in vitro.* However, it is paradoxical that glucocorticoids (GCs) such as dexa lead to bone loss and increased fracture risk in patients undergoing glucocorticoid therapy, causing glucocorticoid-induced osteoporosis (GIOP). In a recent publication, we demonstrated that osteogenic differentiation of progenitor cells isolated from jaw periosteal tissue (JPCs) does not depend on dexa, if the medium is supplemented with human platelet lysate (hPL) instead of fetal bovine serum (FBS). This allows the *in vitro* conditions to be much closer to the natural situation *in vivo* and enables us to compare osteogenic differentiation with and without dexa. In the present study, we demonstrate that the absence of dexa did not reduce mineralization capacity, but instead slightly improved the osteogenic differentiation of jaw periosteal cells. On the other hand, we show that dexa supplementation strongly alters the gene expression, extracellular matrix (ECM), and cellular communication of jaw periosteal cells. The secretome of periosteal cells previously treated with an osteogenic medium with and without dexa was used to investigate the changes in paracrine secretion caused by dexa. Dexa altered the secretion of several cytokines by jaw periosteal cells and strongly induced osteoclast differentiation of peripheral blood mononuclear cells (PBMCs). This study demonstrates how dexa supplementation can influence the outcome of *in vitro* studies and highlights a possible role of periosteal cells in the pathogenesis of glucocorticoid-induced osteoporosis. The methods used here can serve as a model for studying bone formation, fracture healing, and various pathological conditions such as (glucocorticoid-induced) osteoporosis, osteoarthritis, bone cancer, and others, in which the interactions of osteoblasts with surrounding cells play a key role.

## 1 Introduction

Dexamethasone (dexa) is a glucocorticoid drug that is frequently used to treat inflammatory diseases such as rheumatoid arthritis, allergies, and asthma (Boumpas et al., 1993).

Glucocorticoids (GCs) such as dexa act primarily *via* the glucocorticoid receptor, which, after translocation to the nucleus, influences gene expression as a transcription factor. Due to the ubiquitous presence of the GC receptor, GCs act on almost every cell of the body, but through different forms of the receptor, the effects are pleiotropic. The anti-inflammatory effect of dexa is mainly due to the inhibition of the secretion of pro-inflammatory cytokines by cells of the immune system, as well as by preventing the migration of these cells to inflammation sites (Cain and Cidlowski, 2017; Timmermans et al., 2019).

In research laboratories, dexa is used as a cell culture supplement for various purposes. It is frequently used to prevent apoptosis and to promote the proliferation of primary cell cultures, such as bone marrow MSCs, endothelial cells, or hepatocytes (Wen et al., 1997; Moran et al., 2000; Bailly-Maitre et al., 2001; Xiao et al., 2010).

Additionally, dexa is used to stimulate stem cell differentiation. For example, it is used for the differentiation of hepatocytes from embryonic stem cells (ECs) and induced pluripotent stem cells (iPSCs) (Baharvand et al., 2008; Du et al., 2018). It is also used to stimulate myosatellite cells to myoblast differentiation and fusion into myotubes (Syverud et al., 2016). Most frequently, dexa is used to stimulate *in vitro* differentiation of mesenchymal stromal/stem cells (MSCs) toward the adipogenic, chondrogenic, and the osteogenic lineage (Jaiswal et al., 1997; Mackay et al., 1998; Pittenger et al., 1999). In the present work, we focus on the effects of dexa during the osteogenic differentiation of jaw periosteum-derived MSCs (JPCs).

*In vitro* osteogenic differentiation of MSCs is a common tool to study bone development, bone repair, bone-related diseases and drugs, as well as to develop tissue-engineered bone substitutes. The standard method to induce osteogenic differentiation is to incubate MSCs for 2–4 weeks with a medium containing 10% FBS supplemented with ascorbic acid (vitC), β-glycerophosphate (β-gly), and dexamethasone (dexa). While vitC stimulates collagen 1 secretion and β-gly serves as a source of phosphate, dexa is used to stimulate RUNX2 expression, which is a key transcription factor in the early phase of osteoblast differentiation (Nakashima and de Crombrugghe, 2003; Langenbach and Handschel, 2013). However, it is paradoxical that while dexa stimulates osteogenic differentiation *in vitro*, administration of dexa over long periods, or in high doses, leads to bone resorption and osteoporosis in patients (Weinstein, 2012).

This fact points us toward a number of unwanted side effects of dexa supplementation, which can influence experimental outcomes, and impede the examination of MSC behavior during osteogenic differentiation *in vitro*. For example, we made this observation when studying immunomodulatory properties of MSCs from jaw periosteum (JPCs) in co-cultures with immune cells, where the influence of dexa supplementation partially obscured the effect of the co-cultures (Dai et al., 2020; He et al., 2021).

Recently, we demonstrated that dexa supplementation is not necessary to stimulate osteogenic differentiation when using human platelet lysate (hPL) instead of FBS (Wanner et al., 2017). This enabled us to study differences between osteogenic differentiation with and without dexa supplementation.

In the present study, we demonstrate that the omission of dexa does not lead to the deterioration of osteogenic differentiation. Furthermore, we show the influence of dexa on gene expression, ECM composition, and paracrine secretion of jaw periosteal cells and demonstrate the effects of these undesired reactions on the differentiation of osteoclasts.

## 2 Materials and methods

### 2.1 Cell culture

JPCs derived from 12 donors were included in this study in accordance with the local ethical committee (approval number 618/2017BO2) and after obtaining written informed consent. Jaw periosteal tissue was extracted during routine surgery and JPCs were isolated and expanded as previously reported (Umrath et al., 2019). JPCs were grown in hPL5-medium (DMEM/F12 (Gibco) + 5% human platelet lysate (PL BioScience GmbH, Aachen, Germany), 100 U/mL penicillin-streptomycin (Pen-Strep, Lonza, Basel, Switzerland), and 2.5 μg/ml amphotericin B (Biochrom, Berlin, Germany)), and the medium was changed every 2–3 days.

### 2.2 Osteogenic differentiation

To stimulate osteogenic differentiation, JPCs were cultivated in an osteogenic medium (DMEM/F12 + 10% hPL, 100 U/mL Pen-Strep, 2.5 μg/ml amphotericin B, 0.1 mM l-ascorbic acid 2-phosphate (Sigma-Aldrich, St. Louis, MO, United States), and β-glycerophosphate (AppliChem, Darmstadt, Germany)) with and without 4 µM dexamethasone (Sigma-Aldrich, St. Louis, MO, United States). Control samples were cultured in hPL10-medium (DMEM/F12 + 10% hPL, 100 U/mL Pen-Strep, and 2.5 μg/ml amphotericin B). The medium was changed every 2–3 days.

### 2.3 Alizarin Red staining and quantification

After 15 days of osteogenic stimulation, cells were fixed with 4% formalin, and monolayers were stained with 1 ml of Alizarin Red solution (40 mM, pH 4.2) for 20 min. Unbound dye was washed off with distilled water and images were taken using an inverted microscope (Leica, Wetzlar, Germany).

For quantification of bound Alizarin dye, stained plates were incubated with a 10% acetic acid solution for 20 min. Monolayers were detached with a cell scraper and samples were heated at 85°C for 10 min. Subsequently, samples were cooled on ice for 5 min and centrifuged at 20.000 x g for 20 min. Supernatants were neutralized with 10% ammonium hydroxide. Photometrical quantification of alizarin dye was performed at a wavelength of 405 nm.

### 2.4 Gene expression analysis of jaw periosteal cells

RNA isolation from JPCs was performed using the NucleoSpin RNA kit (Macherey-Nagel, Düren, Germany) following the manufacturer’s instructions. RNA concentration was measured using a Qubit 3.0 fluorometer and the corresponding RNA BR Assay Kit (Thermo Fisher Scientific Inc., Waltham, MA, United States). A total amount of 0.5 μg RNA was used for the first-strand cDNA synthesis using the SuperScript Vilo Kit (Thermo Fisher Scientific Inc., Waltham, MA, United States). The quantification of mRNA expression levels was performed using the real-time LightCycler System (Roche Diagnostics, Mannheim, Germany). For the PCR reactions, commercial primer kits (Search LC, Heidelberg, Germany), and DNA Master SYBR Green I (Roche, Basel, Switzerland) were used. The amplification of cDNAs (Table 1) was performed with a touchdown PCR protocol of 40 cycles (annealing temperature between 68 and 58°C), following the manufacturer’s instructions. Copy numbers of each sample were calculated on the basis of a standard curve (standard included in the primer kits) and normalized to the housekeeping gene glyceraldehyde-3-phosphate dehydrogenase (GAPDH).

**TABLE 1:** Genes analyzed by qPCR.

Gene symbol	Gene name
ALPL	Alkaline phosphatase
IBSP (BSP2)	Bone sialoprotein
COMP	Cartilage oligomeric matrix protein
COL1A1	Collagen1α1
COL1A2	Collagen1α2
COL2A1	Collagen2α1
COL7A1	Collagen7α1
COL8A1	Collagen8α1
COL10A1	Collagen10α1
COL11A1	Collagen11α1
COL12A1	Collagen12α1
IL-23	Interleukin 23
IL-27	Interleukin 27
IL-6	Interleukin 6
IL-8	Interleukin 8
LEP	Leptin
LPL	Lipoprotein lipase
BGLAP (OCN)	Osteocalcin
OGN	Osteoglycin
SPARC (OSN)	Osteonectin
SPP1 (OPN)	Osteopontin
TNFRSF11B (OPG)	Osteoprotegerin
SP7 (OSX)	Osterix
POSTN	Periostin
PPARγ	Peroxisome proliferator-activated receptor gamma
TNFSF11 (RANKL)	Receptor activator of nuclear factor kappa B ligand
RUNX2	RUNX family transcription factor 2
SOX9	SRY-box transcription factor 9
TIMP-4	TIMP metallopeptidase inhibitor 4

### 2.5 Protein expression analysis (proteome profiler arrays)

To measure the expression of secreted proteins, supernatants of JPCs cultured for 15 days under untreated (CO) and osteogenic (OB) conditions, with or without dexa were analyzed using proteome profiler array kits (Human Cytokine Array Kit, Human Soluble Receptor Array Kit, and Non-Hematopoietic Panel; R&D Systems, Germany) following the manufacturer’s instructions. Briefly, the membranes were blocked with array buffer for 1 h at room temperature and then incubated with 1.5 ml of sample/array buffer/detection antibody mixtures overnight at 4°C. After washing, the membranes were incubated with 2 ml of diluted streptavidin-HRP at RT for 30 min. After three more washing steps, 1 ml of chemiluminescent reagent mixture was added to the membranes, and luminescence was detected by exposure to radiographic films (GE Healthcare, Chicago, United States) for 10 min. Developed films were scanned, and data analysis of positive signals was carried out using ImageJ software.

### 2.6 Quantification of collagen deposition in monolayers

To detect and quantify collagen in JPC monolayers, JPCs were cultured for 15 days under untreated (CO) and osteogenic conditions, with (OB+D) or without (OB-D) dexa. Then, cells were washed with ddH_2_O and fixed with Bouin liquor (AppliChem, Darmstadt, Germany). Collagen was stained with 0.1% Sirius Red (Sigma-Aldrich, St. Louis, United States) in saturated (1.2%) picric acid solution (AppliChem, Darmstadt, Germany) for 1 h. Wells were washed with 0.01 M HCl and the matrix was dissolved in 1 ml 0.1 M NaOH. 100 µL solution was transferred to a clear 96-well plate and absorption was measured at 550 nm using a microplate reader.

### 2.7 Secretome isolation

175-cm^2^ cell culture flasks were coated with 0.1% gelatin at 37°C for at least 30 min. JPCs were seeded into the coated flasks at a cell density of 1 × 10^6^ cells per flask. The next day medium was changed to osteogenic medium with dexa (OB+D), and without dexamethasone (OB-D) and the medium was changed every 2–3 days. After 10 days of cultivation, 37 ml DMEM/F12 basal medium containing 1% penicillin-streptomycin and 1% amphotericin B was added to the JPCs for exactly 24 h. Then the secretome was collected and centrifuged to remove cell debris. 34 ml supernatant was collected and immediately shock-frozen in liquid N_2_ and stored at −80°C. Later, samples were thawed in a 37 °C water bath and the secretome was concentrated 100-fold by centrifugation using 5 kDa cutoff concentrators (Vivaspin20, Sartorious). The concentrated secretome was stored at −80°C for later use. For osteoclast assays, secretomes of three donors were pooled.

### 2.8 Osteoclast differentiation

Human peripheral blood mononuclear cells (PBMCs) were collected from fresh blood and isolated using gradient centrifugation with Ficoll-Paque PLUS (GE Healthcare, Uppsala, Sweden). PBMCs were resuspended in osteoclast precursor medium (α-MEM containing 10% FBS (Sigma-Aldrich, St. Louis, MO, United States), 1% Pen/Strep, 1% amphotericin b, 20 ng/mL M-CSF (PeproTech, New Jersey, United States)). PBMCs isolated from 15–20 ml blood were seeded in one 75-cm^2^ cell culture flask. Cells were fed with fresh medium every 3 days until they reached 80% confluency. Osteoclast precursors were detached with trypsin and a cell scraper. 6 × 10^4^ cells per well were seeded into 48-well plates with osteoclast precursor medium (negative control) or osteoclast precursor medium with 20 ng/ml RANKL [positive control (PeproTech, New Jersey, United States)]. To test the effect of secretome, cells were treated with osteoclast precursor medium containing 20 ng/ml RANKL +10-fold concentrated secretome (JPC_OB-D/JPC_OB+D). After 6 days of cultivation, cells were fixed and stained for actin [phalloidin-alexa fluor 488 (Biolegend, San Diego, United States)] and nuclei [Hoechst33342 (Promocell, Heidelberg, Germany)]. Images were taken using an Axio Observer Z1 fluorescence microscope (Zeiss, Oberkochen, Germany) equipped with a × 1.25 objective. The number of osteoclasts was quantified using ImageJ software. Osteoclasts were defined as multinucleated cells (≥3 nuclei) with an actin ring. Tartrate-resistant acid phosphatase (TRAP) activity assay was performed using the Acid Phosphatase, Leukocyte (TRAP) Kit (Sigma-Aldrich, St. Louis, MO, United States) following the manufacturer’s instructions.

### 2.9 Gene expression analysis of osteoclasts

Total mRNA extraction from osteoclasts after the 6-day cultivation was performed using the NucleoSpin RNA kit (Macherey-Nagel, Dueren, Germany) according to the manufacturer’s recommendation. 500 ng of RNA was used to synthesize cDNA using LunaScript RT SuperMix Kit (New England Biolabs, Ipswich, MA, United States). mRNA expression levels were quantified with a QuantStudio 3.0 instrument (Thermo Fisher Scientific, Waltham, United States). LUNA universal probe qPCR master mix (New England Biolabs, Ipswich, MA, United States) and PrimeTime qPCR Probe Assay kits (Integrated DNA Technologies, Coralville, Iowa, United States) of indicated genes (GAPDH, CALCR, CTSK, ITGB3, ACP5) were used in the qPCR reactions. The amplification of each experiment was carried out up to 40 circles (95°C 60 s, 95°C 1 s, and 60°C 20 s). Samples were analyzed in triplicates, relative gene expression levels were calculated using the ΔΔC_t_ method, and data are presented as 2^−ΔΔCt^.

### 2.10 Statistical analyses

For the evaluation of calcium quantification, gene expression, protein expression data, collagen quantification, and osteoclast proportion, means ± SEM were calculated and compared by one-way ANOVA (*p* adjusted using Tukey’s multiple comparison test) using GraphPad Prism 8.1.0 software. A *p* value ≤ 0.05 was considered significant.

## 3 Results

### 3.1 Osteogenic differentiation with and without dexamethasone

#### 3.1.1 Jaw periosteal cell mineralization

**FIGURE 1:**
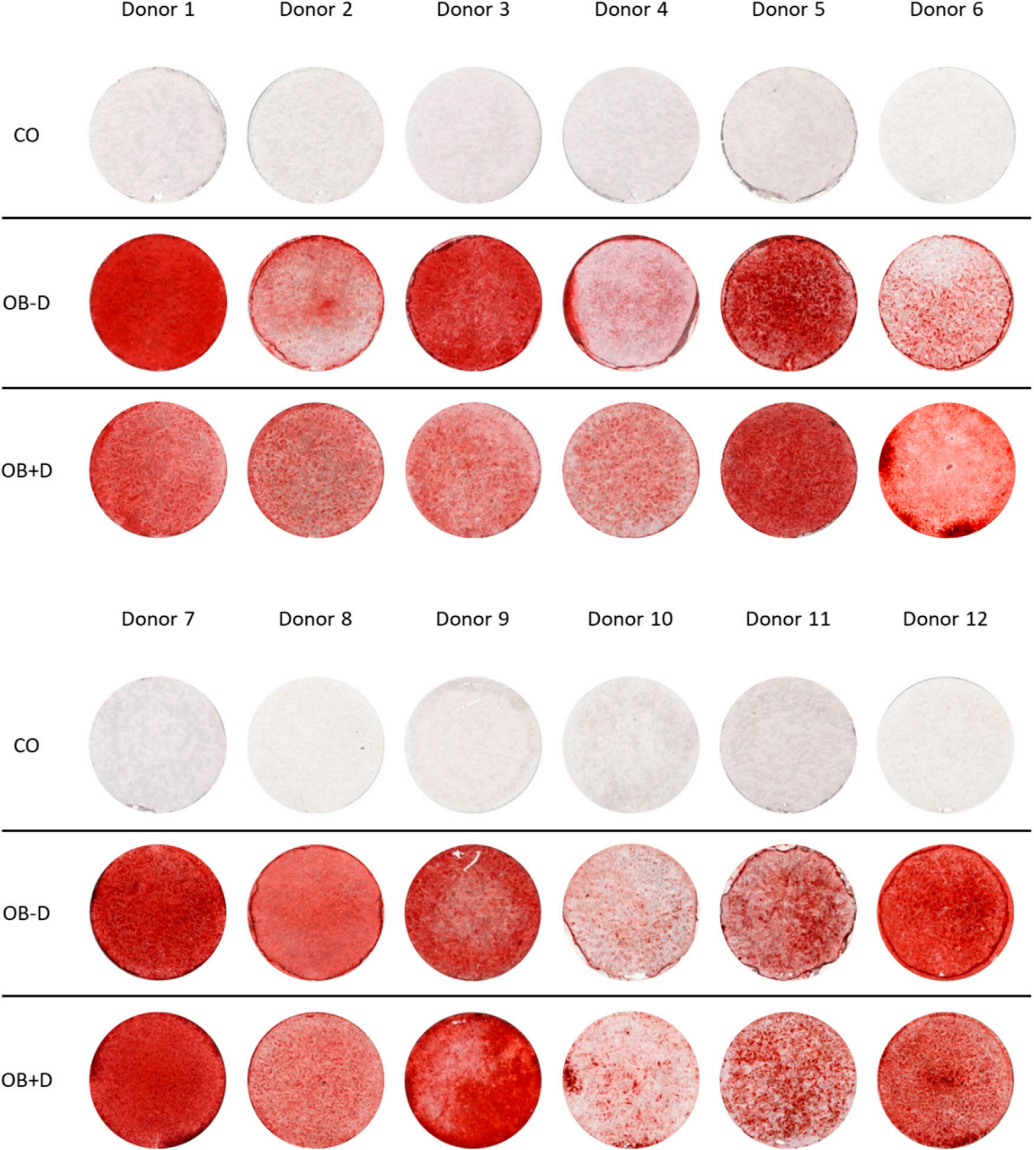
Mineralization of JPCs differentiated *in vitro* with and without dexa. JPCs were cultured for 15 days with control (CO) and osteogenic medium with (OB+D) and without (OB−D) dexamethasone. Calcium phosphate precipitates were detected by Alizarin Red staining.

To compare the osteogenic differentiation of JPCs with and without dexa supplementation, the cells of 12 donors were tested. As shown in Figure 1, we observed considerable differences between the mineralization capacities of cells derived from different donors. However, a major difference in mineralization between cells stimulated with and without dexa was not observed.

**FIGURE 2:**
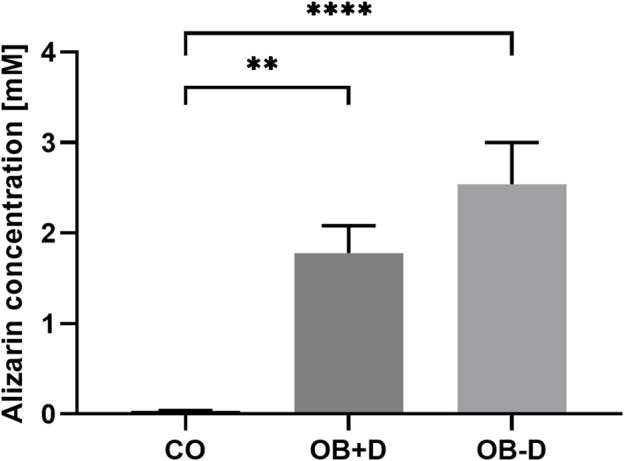
Alizarin quantification of JPCs stimulated with and without dexa. JPCs were cultured for 15 days in control (CO) and osteogenic medium with (OB+D) and without (OB−D) dexa. Calcium phosphate precipitates were stained with Alizarin dye. Bound dye was solubilized and quantified photometrically. Mean values ± SEM were calculated and compared using one-way ANOVA and Tukey’s multiple comparison test (n = 9, ** = *p*< 0.01, **** = *p*< 0.0001).

After Alizarin Red staining, calcium phosphate precipitates were photometrically quantified (Figure 2). On average, we found stronger mineralization of JPCs cultured without dexa (OB-D, 2.54 ± 0.46 mM) compared with JPCs with dexa (OB+D, 1.78 ± 0.36 mM). However, differences between OB+D and OB-D conditions were not statistically significant, due to high donor variations. The differences between calcium concentrations in untreated (CO) and osteogenically stimulated cells reached a higher level of significance under osteogenic conditions without dexa (OB-D) (*p* < 0.0001) than under OB+D conditions (*p* < 0.0022).

These results indicate that dexa supplementation of the osteogenic medium does not provide a benefit for jaw periosteal cell mineralization.

#### 3.1.2 Expression of osteogenic marker genes by jaw periosteal cells

The tendency of a higher mineralization of JPCs in an osteogenic medium without dexa is also reflected in a higher expression of osteogenic marker genes.

**FIGURE 3:**
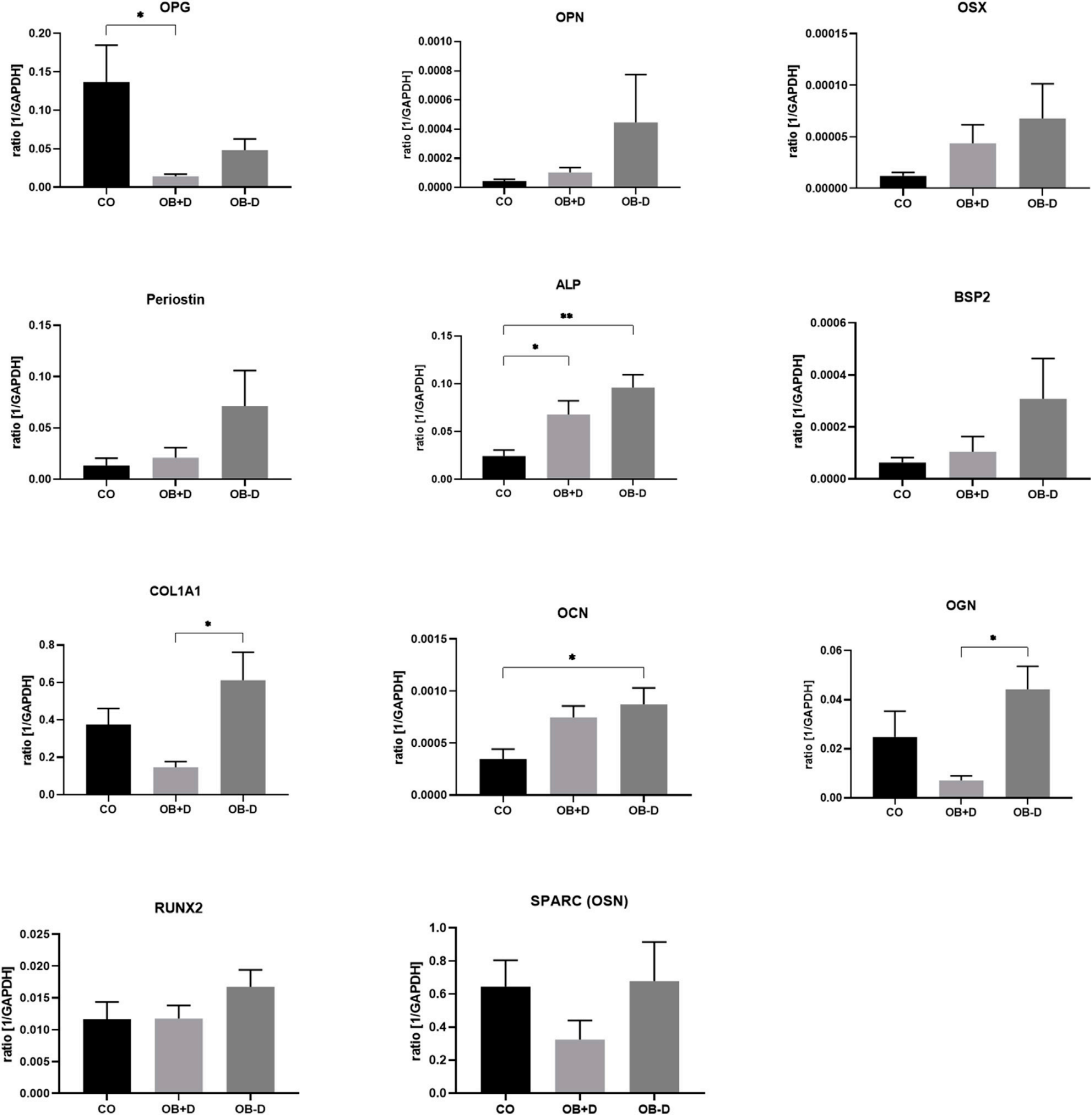
Expression of osteogenic marker genes by JPCs cultured for 15 days with control (CO) and osteogenic medium with (OB+D) and without dexa (OB−D). Mean ± SEM values of mRNA copy numbers normalized by the housekeeping gene (GAPDH) were calculated and compared using one-way ANOVA and Tukey’s multiple comparison test (n = 7, * = *p*< 0.05, ** = *p*< 0.01).

As shown in Figure 3 and Supplementary Table S1, the average expression of ALPL, OCN, RUNX2, COL1A1 and all other tested genes is higher in JPCs treated with osteogenic medium without dexa (OB−D). However, with the exception of COL1A1 (*p* = 0.0111) and OGN (*p* = 0.0132), no statistical significance between OB+D and OB−D groups could be detected due to high donor variations.

#### 3.1.3 Expression of chondrogenic and adipogenic marker genes

Chondrogenic and adipogenic marker gene expression was analyzed to determine the effects of dexa on the regulation of these pathways under osteogenic conditions.

**FIGURE 4:**
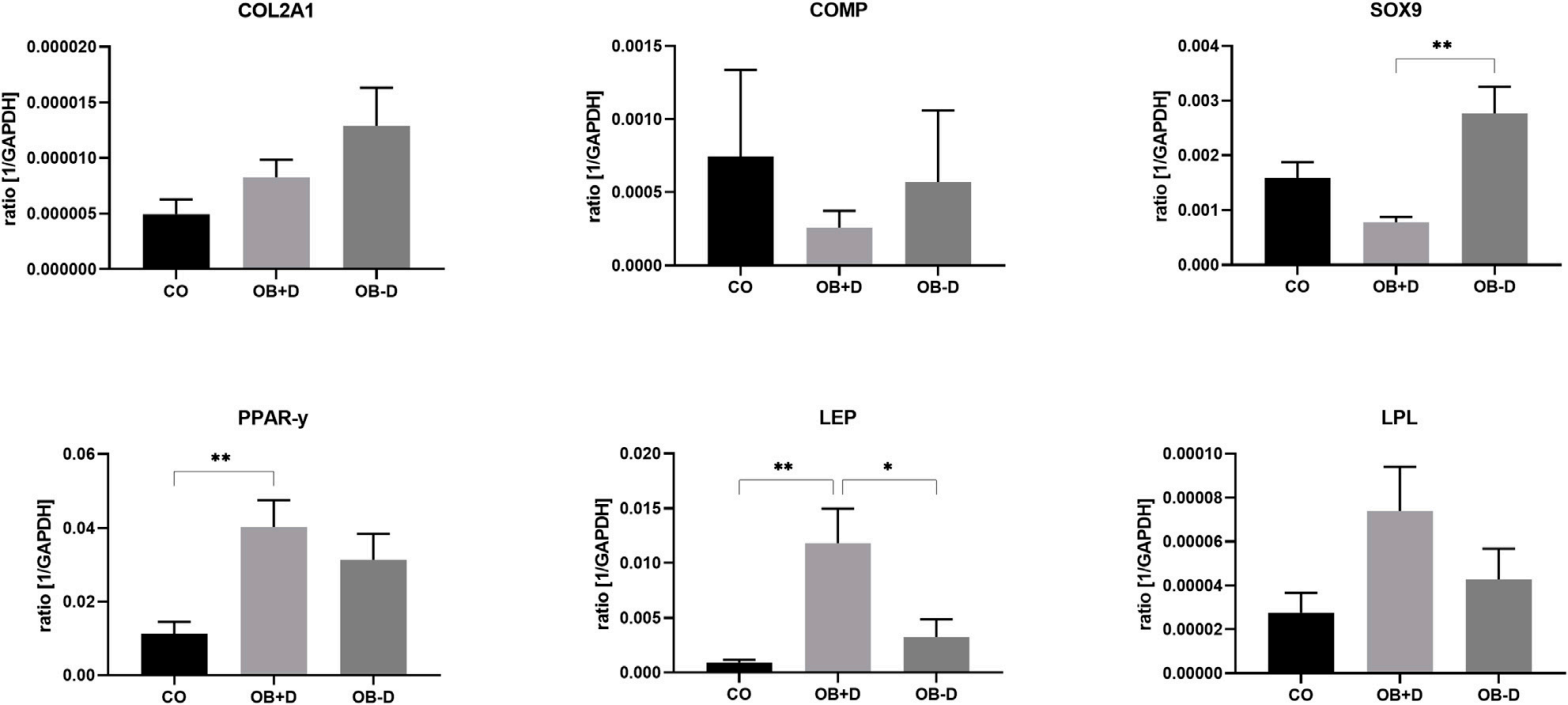
Expression of chondrogenic marker genes (upper panel) and adipogenic marker genes (lower panel) by JPCs cultured for 15 days with control medium (CO) and osteogenic medium with (OB+D) and without (OB−D) dexa. Mean ± SEM values of mRNA copy numbers normalized by the housekeeping gene (GAPDH) were calculated and compared using one-way ANOVA and Tukey’s multiple comparison test (n = 7, * = *p*< 0.05, ** = *p*< 0.01).

As shown in Figure 4 and Supplementary Table S2, a higher expression of chondrogenic marker genes COL2A1, COMP, and SOX9 was observed during osteogenic differentiation without dexa (OB-D) compared with differentiation with dexa (OB+D). SOX9, which is a major transcription factor during chondrogenesis, was expressed 3.6-fold stronger in the OB−D group compared with the OB+D group (*p* = 0.0013). In contrast, adipogenic marker genes PPARγ, LEP and LPL were induced during osteogenic differentiation in the OB+D group. Significantly higher expression in OB+D compared with OB−D samples was detected for LEP (*p* = 0.0217), which encodes a hormone typically secreted by adipocytes.

### 3.2 Alterations in cell adhesion

**FIGURE 5:**
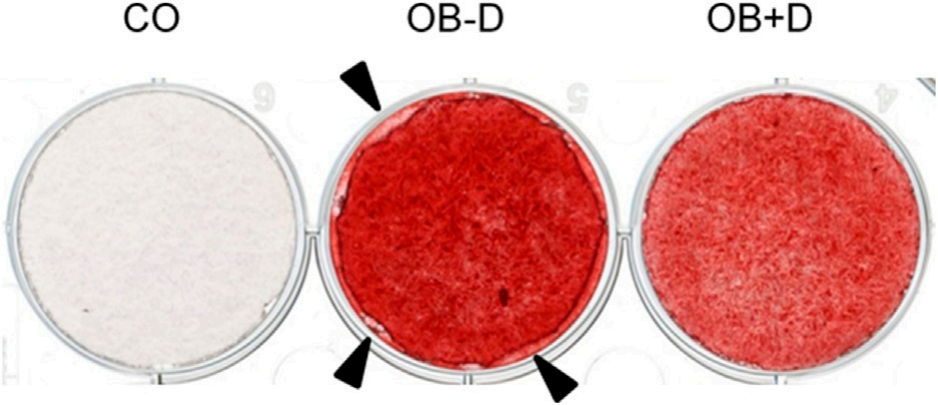
Alterations in cell adhesion of JPCs. JPCs were cultured for 15 days with control medium (CO) and osteogenic medium with (OB+D) and without (OB−D) dexa. Cell mineralization was stained with the Alizarin Red dye. Detachment of the cell layer in the OB-D group is indicated by black arrows.

While mineralization seemed to be slightly improved in osteogenic medium without dexa, we observed differences in the adhesion of cells treated with and without dexa. JPCs incubated without dexa during osteogenic differentiation showed reduced plastic adherence and detachment of cell monolayers, as shown in Figure 5. We attribute these changes in cell adhesion to changes in the composition of the ECM, which we examine in more detail in the following section.

#### 3.2.1 Collagen deposition

**FIGURE 6:**
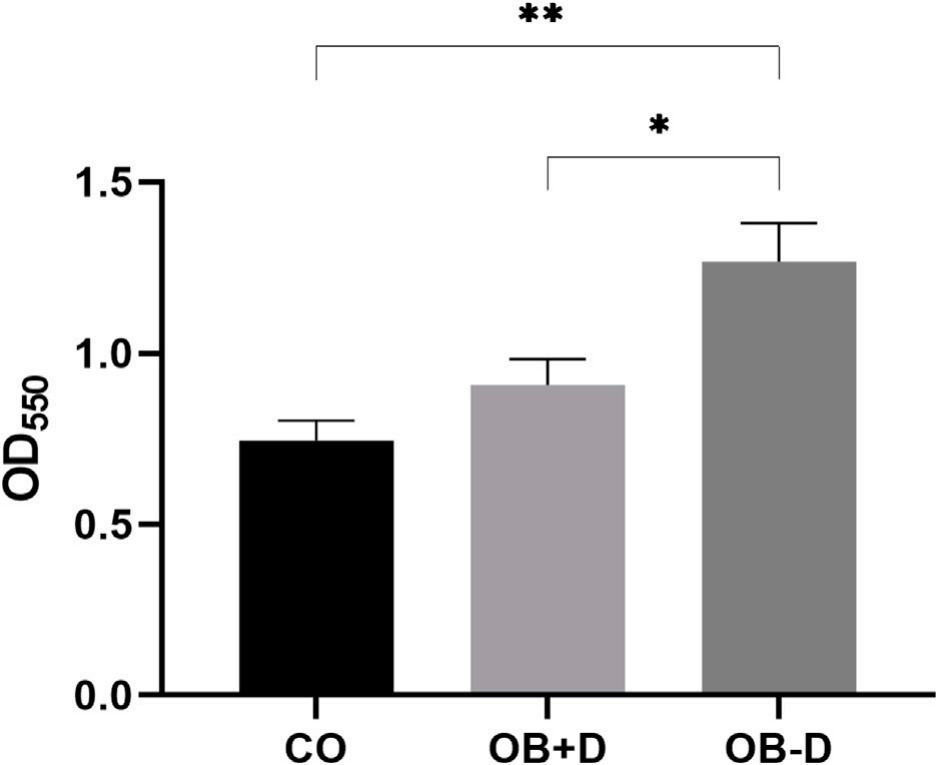
Quantification of collagen deposition. JPCs were cultured for 15 days with control medium (CO) and osteogenic medium with (OB+D) and without (OB−D) dexa. Collagen within monolayers was stained with picrosirius red, and intensities were quantified by measuring absorption at 550 nm. Mean ± SEM values of OD550 were calculated and compared using one-way ANOVA and Tukey’s multiple comparison test (n = 5, * = *p* < 0.05, ** = *p* < 0.01).

To demonstrate the effect of dexa on ECM composition, collagens were stained and quantified using picrosirius red staining (Figure 6).

Picrosirius red staining specifically binds to collagen in tissues and can be used to quantify collagen deposition *in vitro* (Williams et al., 2001). Cells treated with osteogenic medium without dexa (OB−D) showed significantly higher collagen deposition compared with the OB+D group (*p* = 0.0295) and the control group (*p* = 0.0027).

#### 3.2.2 Expression of extracellular matrix-related genes

**FIGURE 7:**
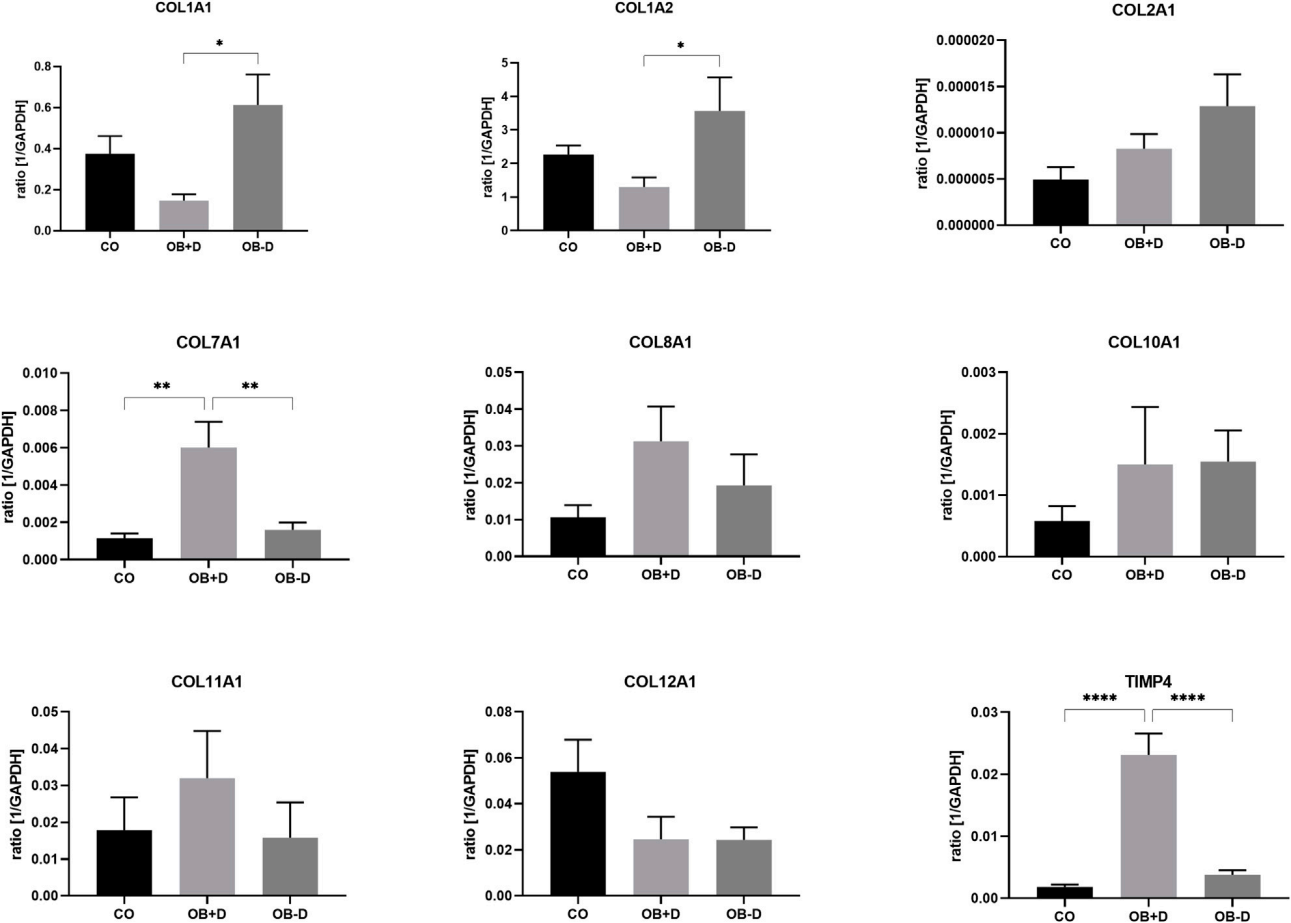
Expression of ECM-related genes by JPCs cultured for 15 days with control medium (CO) and osteogenic medium with (OB+D) and without (OB−D) dexa. Mean ± SEM values of mRNA copy numbers normalized by the housekeeping gene (GAPDH) were calculated and compared using one-way ANOVA and Tukey’s multiple comparison test (n = 7, * = *p*< 0.05, ** = *p*< 0.01, **** = *p* < 0.0001).

Changes in adherence during osteogenic differentiation with and without dexa are also reflected in the expression of matrix-related genes (Figure 7; Supplementary Table S3).

While COL1A1 (*p* = 0.0111), COL1A2 (*p* = 0.0464), and COL2A1 were upregulated during osteogenic differentiation without dexa (OB−D), COL7A1, COL8A1, COL11A1, and TIMP-4 were upregulated in OB+D samples. Especially COL7A1 (*p* = 0.0045) and TIMP-4 (*p* < 0.0001) were significantly induced by dexa supplementation compared with osteogenic stimulation without dexa.

ECM-related and other proteins secreted by JPCs were analyzed by protein arrays (Supplementary Figures S1–6).

### 3.3 Paracrine secretion of osteoclastogenic factors by jaw periosteal cells

#### 3.3.1 Expression of osteoclastogenesis-related genes

**FIGURE 8:**
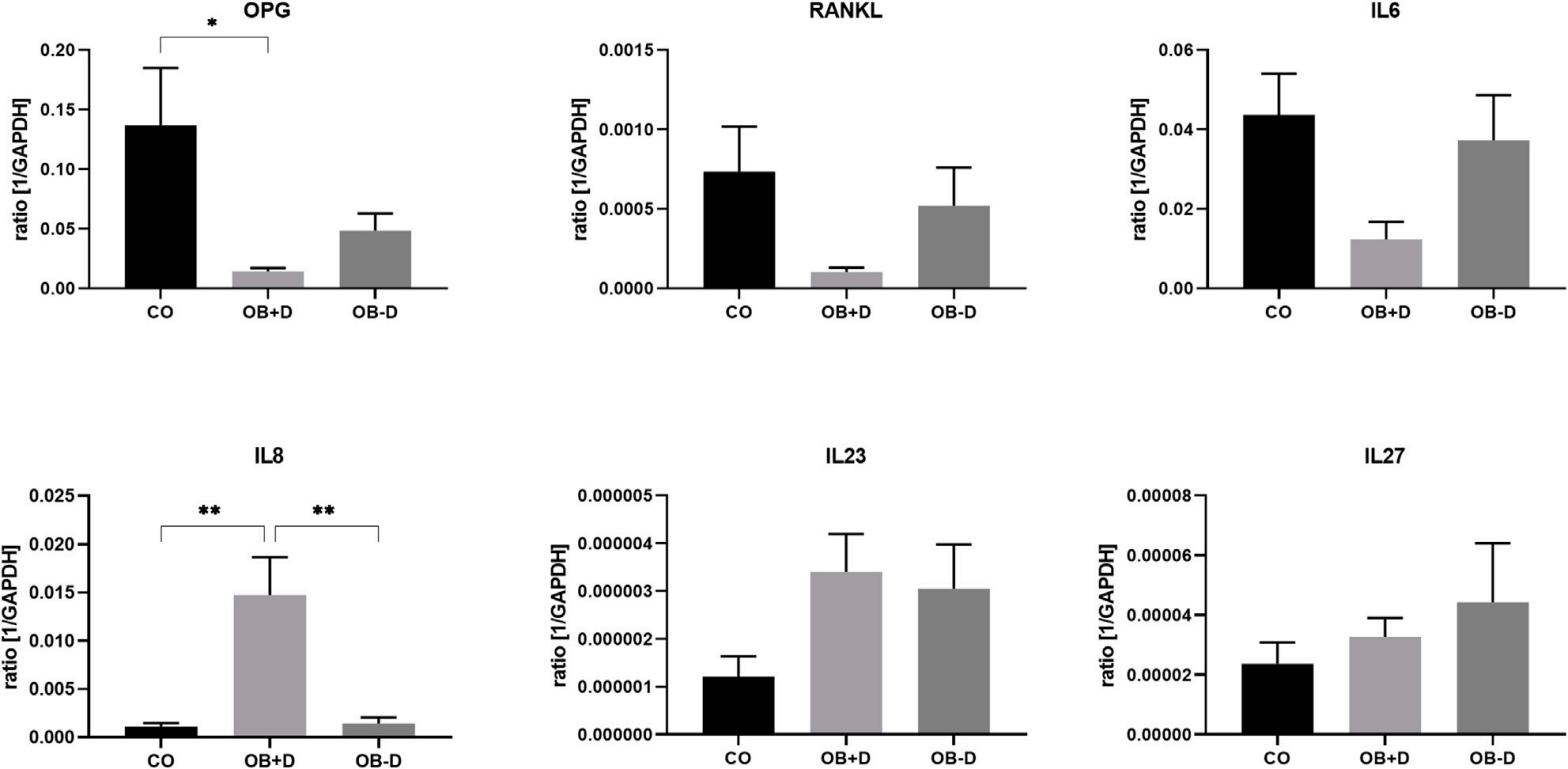
Expression of osteoclastogenesis-related genes by JPCs cultured for 15 days with control medium (CO) and osteogenic medium with (OB+D) and without (OB−D) dexa. Mean ± SEM values of mRNA copy numbers normalized to the housekeeping gene GAPDH were calculated and compared using one-way ANOVA and Tukey’s multiple comparison test (n = 7, * = *p*< 0.05, ** = *p*< 0.01).

To study the effect of dexa supplementation on cellular interactions of JPCs with other relevant cell types, the expression of genes involved in osteoclast differentiation was analyzed in JPCs treated with osteogenic medium with and without dexa (Figure 8, Supplementary Table S4).

As shown in Figure 8, IL-8, which stimulates osteoclast differentiation, was highly induced in OB+D samples (CO vs. OB+D, *p* = 0.0014; OB−D vs. OB+D, *p* = 0.0013). On the other hand, OPG, which inhibits osteoclast differentiation, was downregulated. IL-6, which is commonly known as inducer of osteoclastogenesis was downregulated by dexa (CO vs. OB+D, *p* = 0.0311). IL-23, another inducer of osteoclastogenesis was upregulated in both OB+D and OB−D samples. The expression of IL-27, an inhibitor of osteoclast differentiation, was not relevantly affected by dexa.

#### 3.3.2 Secretion of osteoclastogenesis-related factors

**FIGURE 9:**
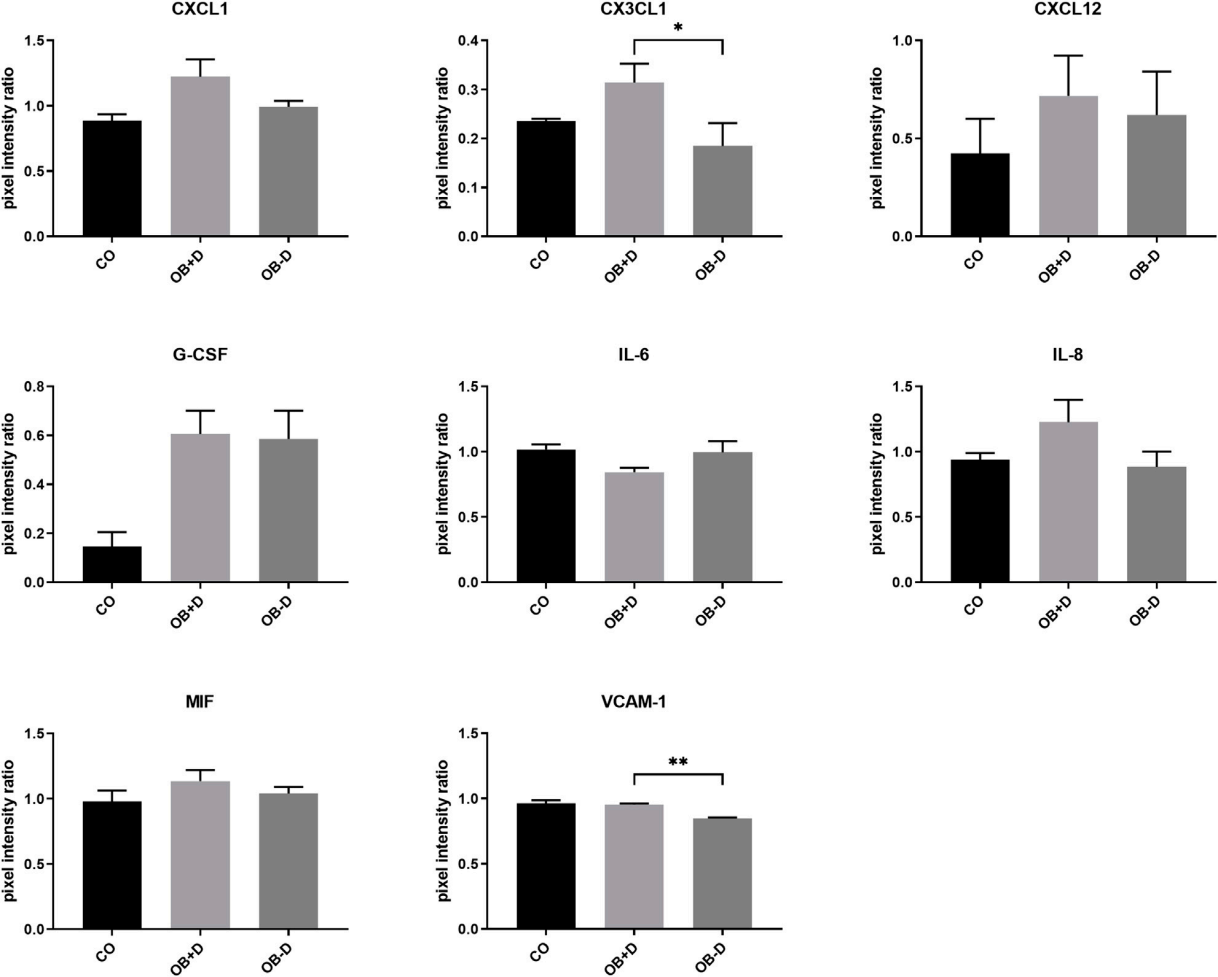
Secretion of osteoclastogenesis-related proteins by JPCs cultured for 15 days with control medium (CO) and osteogenic medium with (OB+D) and without (OB−D) dexa. Protein expression was examined by detection of soluble factors secreted into culture supernatants using proteome profiler arrays and analysis of pixel intensities of detected signals using ImageJ software. Pixel intensity ratio mean ± SEM values were calculated by normalization to internal controls and compared using one-way ANOVA and Tukey’s multiple comparison test (n = 3, * = *p*< 0.05, ** = *p* < 0.01).

Secretion of soluble factors was analyzed using antibody arrays. Data analysis (Figure 9) showed a higher expression of numerous proteins associated with osteoclast differentiation such as IL-8, CX3CL1, CXCL1, CXCL12, MIF, and VCAM-1 in supernatants of JPCs stimulated with osteogenic medium containing dexa (OB+D). However, with exception of CX3CL1 (*p* = 0.0239) and VCAM-1 (*p* = 0.0062) differences were not statistically significant between OB−D and OB+D groups. Interestingly, IL-6, which is also known to stimulate osteoclast differentiation (Amarasekara et al., 2018), was slightly downregulated in OB+D samples.

#### 3.3.3 Effects of jaw periosteal cells’ secretome on the osteoclast differentiation of peripheral blood mononuclear cells

To analyze the effect of dexa on the secretion of osteoclastogenic factors by JPCs during osteogenic differentiation, PBMCs were treated with secretome isolated from JPCs after 10 days of osteogenic stimulation with (JPC_OB+D) and without (JPC_OB−D) dexa.

PBMCs were isolated using gradient centrifugation and stimulated for 6 days with M-CSF. Then the cells were seeded into 48-well plates and treated for 6 days with M-CSF (negative control), M-CSF + RANKL (20 ng/ml) (positive control), M-CSF + RANKL +10x JPC-secretome -Dexa (JPC_OB−D), and M-CSF + RANKL +10x JPC-secretome + Dexa (JPC_OB+D).

**FIGURE 10:**
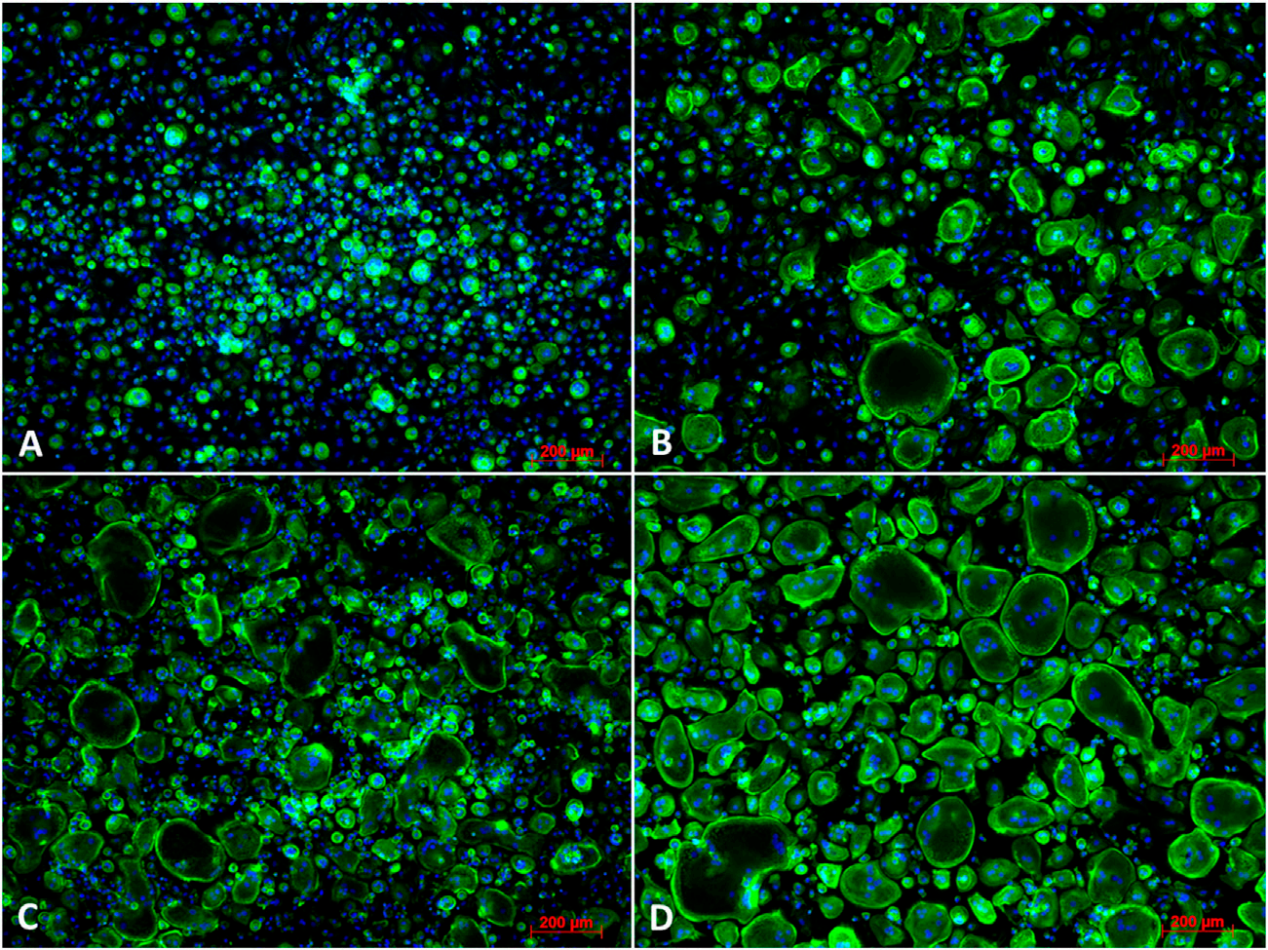
Fluorescence images of osteoclast differentiation. PBMCs were incubated **(A)** with M-CSF (negative control); **(B)** with M-CSF + RANKL (positive control); **(C)** with M-CSF + RANKL + secretome (10x) of JPCs incubated with osteogenic medium without dexa (JPC_OB−D); **(D)** with secretome (10x) of JPCs incubated with osteogenic medium with dexa (JPC_OB+D). Cells were incubated with different media supplementations for 6 days and then fixed and stained for actin (phalloidin-alexa fluor 488) and nuclei (Hoechst). Scale bars = 200 µm.

**FIGURE 11:**
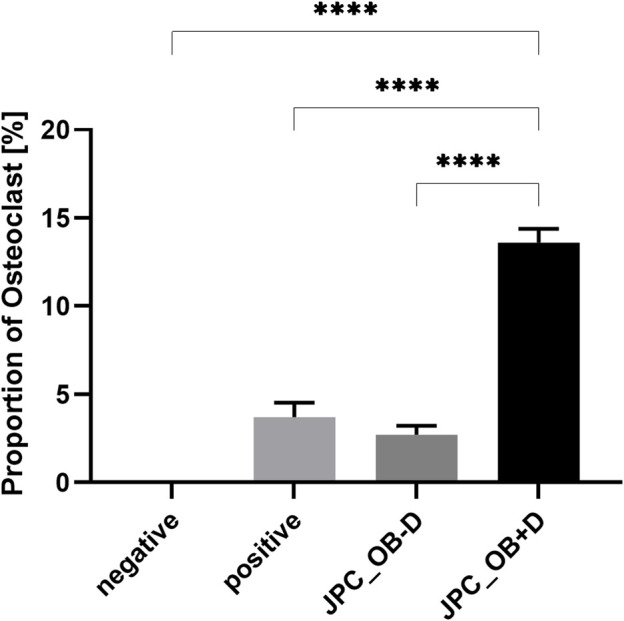
Quantification of mature osteoclasts in the overall cell population. M-CSF + RANKL stimulated PBMCs were incubated with secretome (10x) of JPCs treated with osteogenic medium with dexa (JPC_OB+D) and without dexa (JPC_OB−D). Controls were treated with M-CSF (negative) and M-CSF+RANKL (positive). After 6 days cells were fixed and stained for actin (phalloidin-alexa fluor 488) and nuclei (Hoechst). Microscopic images were analyzed using ImageJ software. Cells were defined as osteoclasts when having three or more nuclei and showing an actin ring. Means ± SEM of osteoclast proportions were calculated and compared using multiple t-tests (n = 3, ****=*p* < 0.0001).

As shown in Figure 10, osteoclast differentiation occurred in the positive control group and in groups supplemented with either JPC secretomes (JPC_OB−D/JPC_OB+D). However, the number of mature osteoclasts in the group treated with JPC_OB+D secretome (D) was markedly higher than in the group treated with JPC_OB−D secretome (C). The number of osteoclasts per well (actin ring and ≥3 nuclei) was counted and the proportions of osteoclasts to total cell numbers were calculated. As shown in Figure 11, the proportion of multinucleated osteoclasts was significantly higher in the group of PBMCs treated with secretome from JPCs previously stimulated with dexa (JPC_OB+D) compared with all other groups. In contrast, osteoclast proportion was reduced in the group treated with JPC_OB−D secretome compared with the positive control.

**FIGURE 12:**
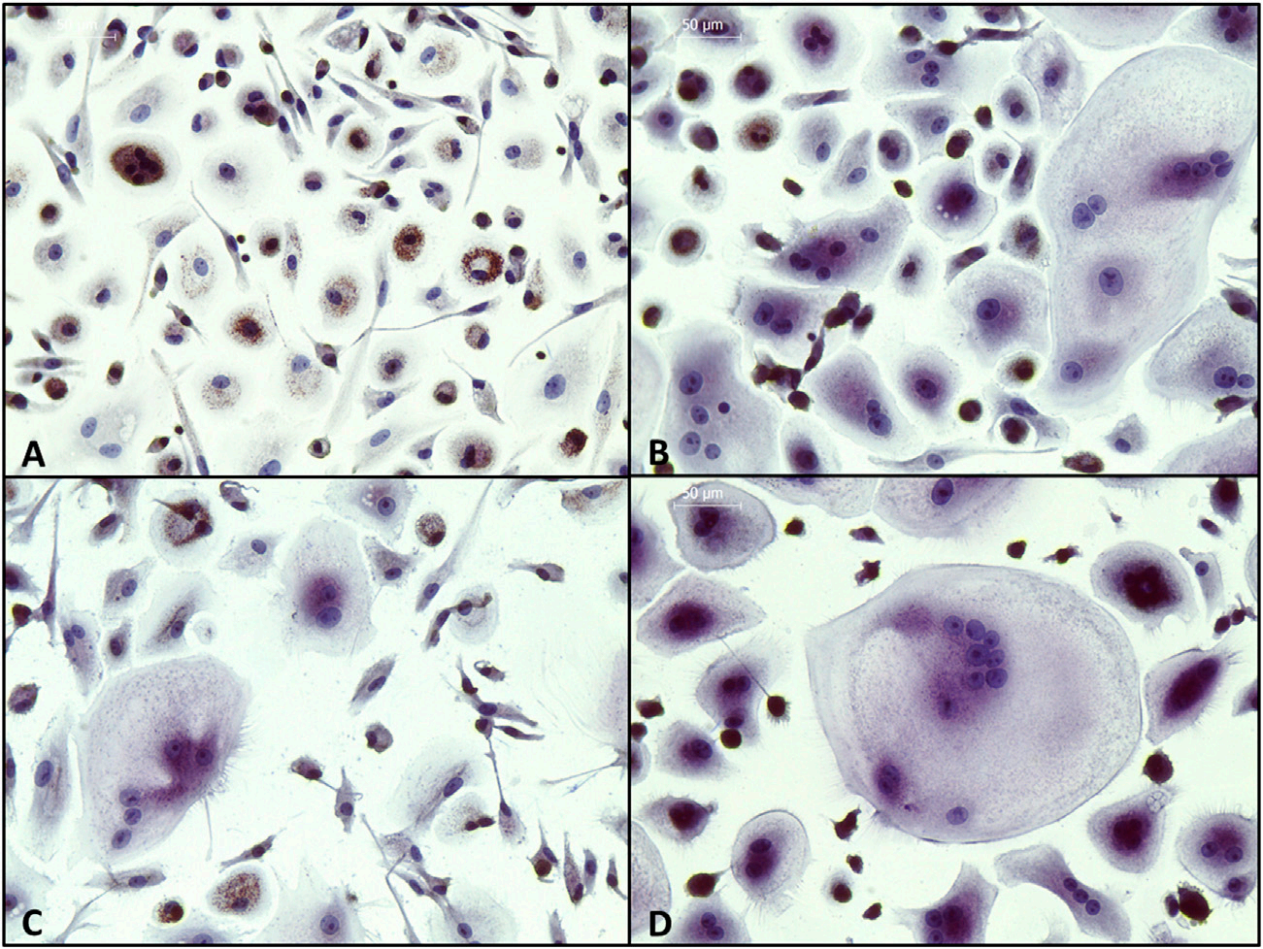
Detection of tartrate-resistant acid phosphatase (TRAP) in PBMCs. Cells were incubated (A) with M-CSF (negative control); (B) with M-CSF + RANKL (positive control); (C) with M-CSF + RANKL + secretome (10x) of JPCs incubated with osteogenic medium without dexa (JPC_OB-D); (D) with secretome (10x) of JPCs incubated with osteogenic medium with dexa (JPC_OB+D). Cells were incubated with different media supplementations for 8 days and then fixed and stained for TRAP (purple) and nuclei (blue). (Scale bars = 50 μm).

Tartrate-resistant acid phosphatase (TRAP) activity was detected by staining with Naphthol AS-BI phosphoric acid and Fast Garnet GBC, and nuclei were counterstained with hematoxylin. As shown in Figure 12, osteoclasts were stained positive for TRAP activity (purple) and showed multiple nuclei (blue) in secretome-treated samples as well as in the positive control, while no activity and no multinucleated cells were detected in the negative control.

##### 3.3.3.1 Expression of osteoclast marker genes by peripheral blood mononuclear cells

**FIGURE 13:**
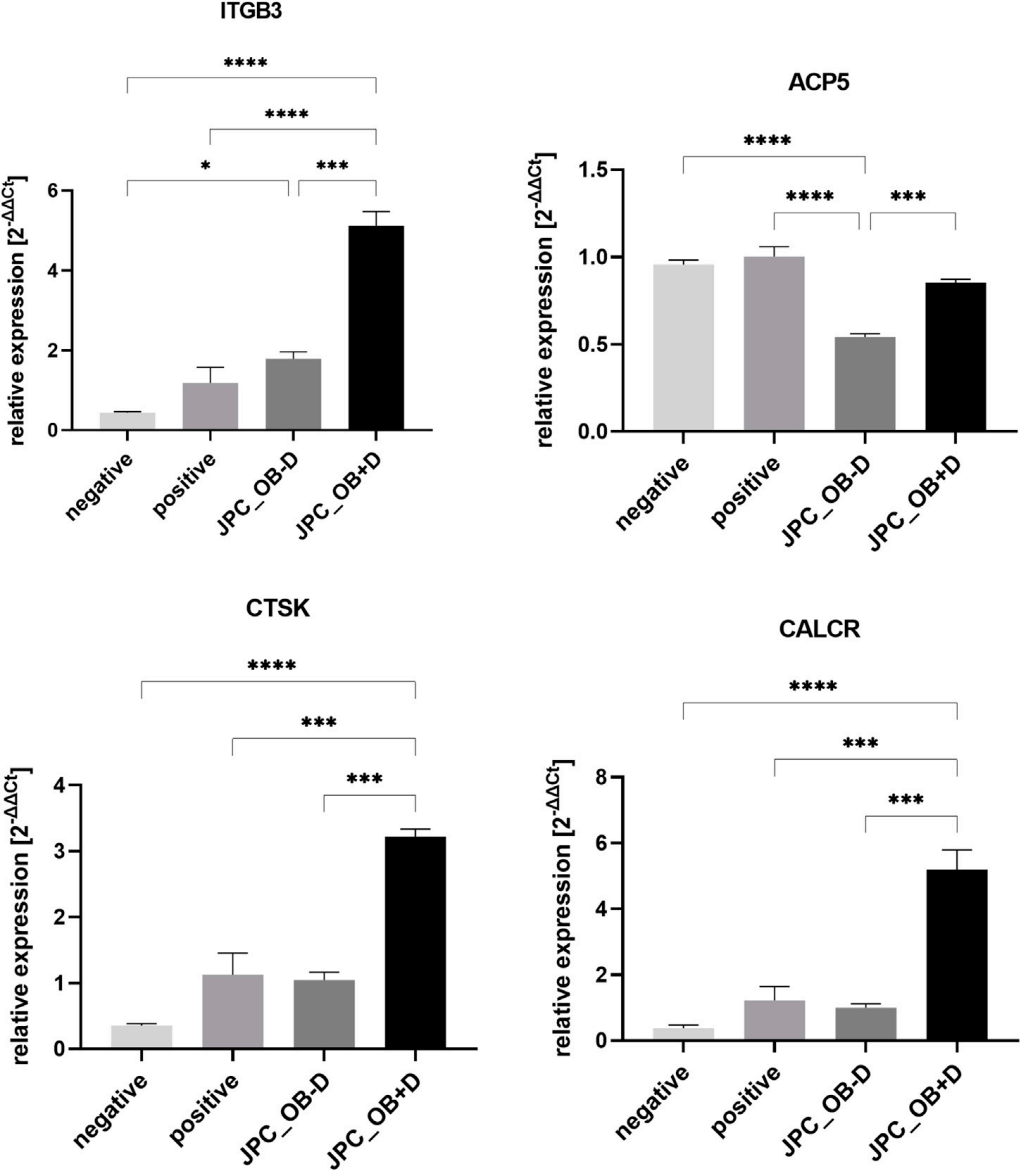
Relative expression of osteoclast marker genes ITGB3, ACP5 (TRAP), CTSK and CALCR. M-CSF + RANKL stimulated PBMCs were incubated with secretome (10x) of JPCs treated with osteogenic medium with dexa (JPC_OB+D) and without dexa (JPC_OB-D). Controls were treated with M-CSF (negative) and M-CSF+RANKL (positive). After 6 days RNA was isolated and gene expression was analyzed by qPCR. ΔCt values were calculated using the housekeeping gene GAPDH as endogenous reference. Gene expression induction (2^-ΔΔCt^) was calculated relative to the positive control. The different treatments were compared using one-way ANOVA and Tuckeys multiple comparison test (*n* = 3, * = *p* < 0.05, *** = *p* < 0.001, **** = *p* < 0.0001).

Expression of osteoclast marker genes by PBMCs showed significantly higher levels of all tested genes (ITGB3, *p* = 0.0001; ACP5 (TRAP), *p* = 0.0007; CTSK, *p* = 0.0001; CALCR, *p* = 0.0002) in the group treated with secretome from dexa-treated JPCs (JPC_OB+D) compared with JPCs stimulated without dexa (JPC_OB-D) (Figure 13; Supplementary Table S5). Interestingly, ACP5 (TRAP) expression was downregulated in the JPC_OB−D and JPC_OB+D group compared with the positive and negative control. However, downregulation compared with positive and negative control was not significant in the JPC_OB+D group but highly significant in the JPC_OB−D group (JPC_OB−D vs. negative, *p* = <0.0001; JPC_OB−D vs. positive, *p* = <0.0001).

Relative expression of osteoclast marker genes ITGB3, ACP5 (TRAP), CTSK, and CALCR. M-CSF + RANKL stimulated PBMCs were incubated with secretome (10x) of JPCs treated with osteogenic medium with dexa (JPC_OB+D) and without dexa (JPC_OB−D). Controls were treated with M-CSF (negative) and M-CSF + RANKL (positive). After 6 days RNA was isolated and gene expression was analyzed by qPCR. ΔC_t_ values were calculated using the housekeeping gene GAPDH as endogenous reference. Gene expression induction (2^−ΔΔCt^) was calculated relative to the positive control. The different treatments were compared using one-way ANOVA and Tukey’s multiple comparison test (n = 3, * = *p*< 0.05, *** = *p*< 0.001, **** = *p*< 0.0001).

## 4 Discussion

### 4.1 Osteogenic differentiation with and without dexamethasone

Dexamethasone supplementation of cell culture media is standard to stimulate adipogenic, chondrogenic, and osteogenic differentiation of MSCs *in vitro* (Robert et al., 2020). However, effects of dexa *in vivo* are quite different than what can be observed *in vitro*. This becomes very obvious when studying osteogenic differentiation *in vitro*, where dexa is used to induce osteogenesis, while dexa administration *in vivo* can cause glucocorticoid-induced osteoporosis (GIOP). In the present study, we demonstrated that *in vitro* cultured JPCs under human platelet lysate supplementation showed a response to dexa that may indicate the underlying mechanism of clinical observations after glucocorticoid administration. We found that dexa supplementation strongly affected all aspects of periosteal cell behavior we examined (osteogenic differentiation, extracellular matrix, secretome, interaction with osteoclasts) *in vitro*.

Because of considerable donor variations in the osteogenic potential of JPCs, irrespective of dexa supplementation, we included a larger number of donors in this study (Figure 1). Unexpectedly, we found a tendency towards stronger mineralization without dexa supplementation (Figure 2). This result is also supported by the analysis of 11 osteogenic marker genes, all of which were in tendency higher expressed during osteogenic differentiation without dexa than in the presence of dexa (Figure 3). Especially COL1A1, the major organic component of bone matrix and OGN, which was found to be downregulated in senile osteoporosis (Chen et al., 2017), was significantly higher expressed in osteogenically treated JPCs without dexa. Some of the analyzed genes (OPG, OGN, COL1A1, and SPARC) were even downregulated under dexa supplementation compared with the untreated controls. Most significantly OPG, which inhibits osteoclastogenesis as a decoy receptor for RANKL (Boyce and Xing, 2008), was significantly downregulated compared with the untreated control.

Furthermore, the analysis of chondrogenic and adipogenic marker genes during osteogenic differentiation revealed a higher expression of chondrogenic markers without dexa, while the expression of adipogenic markers was increased by dexa supplementation (Figure 4).

A higher expression of chondrogenic markers is in accordance with the close relationship of chondrogenesis and osteogenesis, and simultaneous regulation of signaling pathways during endochondral ossification (Jing et al., 2017). This observation further indicates, that chondrogenic differentiation protocols, which also include dexa supplementation, might as well be improved by omitting dexa.

In contrast to chondrogenic differentiation, adipogenic differentiation is known to antagonize osteogenic pathways (James, 2013; Yuan et al., 2016). Thus, induction of adipogenesis-related genes in JPCs by dexa may explain the weaker mineralization and expression of osteogenic markers that we observed.

Together, these data prove that osteogenic differentiation is at least as effective without dexa as with dexa when the medium is supplemented with hPL.

### 4.2 Extracellular matrix changes by dexamethasone supplementation

During osteogenic differentiation without dexa, we observed that cell monolayers detached at the edge of the wells (Figure 5), which we did not observe when JPCs were treated with dexa. We concluded that dexa also alters ECM composition. To support this hypothesis, we analyzed collagen deposition by picrosirius red staining and quantification (Figure 6). We found a significantly higher deposition of collagen in wells treated with osteogenic medium without dexa compared with wells treated with dexa.

Analysis of ECM-related genes showed a significant downregulation of COL1A1 and COL1A2 by dexa (Figure 7). Instead, other types of collagen (COL7A1, COL8A1, and COL11A1) were upregulated. Most significantly the upregulation of COL7A1, which builds anchoring fibrils between epithelia and stroma (Burgeson et al., 1985), could explain better adherence in dexa-treated wells. Furthermore, TIMP-4, a soluble inhibitor of matrix metalloproteinases (MMPs), was significantly upregulated by dexa treatment of JPCs (Figure 7). TIMP-4 inhibits MMP-2, which plays an important role in bone remodeling (Hardy and Fernandez-Patron, 2020). Inactivation mutation of MMP-2 causes Winchester Syndrome which is associated with arthropathy, osteoporosis, and even osteolysis of carpal and tarsal bones (Chen, 2017). Therefore, inhibition of MMP-2 by TIMP-4 might have similar effects on bone remodeling, and thereby bone stability. Significantly higher protein levels of TIMP-4 were also detected in cell culture supernatants of JPCs stimulated with osteogenic medium containing dexa (Figure 7). MMP-2 protein concentration was not affected by dexa (supplementary material).

### 4.3 Effect of dexamethasone treatment of jaw periosteal cells on osteoclast differentiation

In previous coculture studies, we found clear effects of dexa on interactions of JPCs with dendritic cells and macrophages during osteogenic differentiation that were independent of the osteogenic stimulation (Dai et al., 2020; He et al., 2021). In order to investigate whether dexa-treated JPCs also elicit pro- or anti-osteoclastogenic effects, we analyzed paracrine effects of dexa treatment during osteogenic differentiation of JPCs on the differentiation of PBMCs to osteoclasts. To exclude direct effects of dexa and hPL, we used secretome of JPCs isolated in DMEM/F12 basal medium and concentrated it using 5 kDa cutoff filter columns (Martins et al., 2017). We observed a highly significant increase in osteoclast numbers using secretome of dexa-treated JPCs (JPC_OB+D) compared with all other groups (Figure 11). Compared with the positive control, stimulated with M-CSF/RANKL, PBMCs showed 3.7-times higher osteoclast numbers in the presence of the secretome isolated from dexa-treated JPCs. In contrast, osteoclast differentiation in samples treated with secretome of cells treated without dexa was slightly reduced by 0.27-fold, however without reaching statistical significance.

These results might also be relevant concerning the growing interest in therapeutic applications of MSC secretome or exosomes (Codispoti et al., 2018; Wechsler et al., 2021).

Gene expression analysis of typical osteoclast marker genes in PBMCs also demonstrated the osteoclastogenic function of dexa-treated JPCs. Expression of ITGB3, ACP5 (TRAP), CTSK, and CALCR was significantly higher in PBMCs treated with secretome from dexa-treated JPCs (JPC_OB+D) compared with secretome from JPCs stimulated without dexa (JPC_OB−D) (Figure 13). Furthermore, ACP5 (TRAP) expression was significantly downregulated in the JPC_OB−D group, indicating an anti-osteoclastogenic effect of JPCs osteogenically stimulated without dexa. Due to the limited number of targets on the protein arrays used here, no precise conclusions can be drawn about the factors mediating osteoclastogenesis. Furthermore, in most cases no significant differences between OB−D and OB+D groups could be detected.

However, we observed a significant downregulation of osteoprotegerin (OPG), a decoy receptor for RANKL, in dexa-treated JPCs (Figure 8). Also, gene expression and secretion of IL-6 which is usually regarded as inducer of osteoclastogenesis were in tendency downregulated by dexa (Amarasekara et al., 2018). However, it has also been shown that IL-6 directly inhibits osteoclastogenesis by suppression of RANKL signaling in osteoclast precursors (Blanchard et al., 2009). Furthermore, we detected a higher secretion of a number of cytokines (IL-8, MIF, CXCL1, CX3CL1, and CXCL12) by OB+D-treated JPCs. However, with exception of CX3CL1, without statistical significance (Figure 9). All of these cytokines have previously been associated with the promotion of osteoclastogenesis, bone resorption, or bone metastasis (Bendre et al., 2003; Grassi et al., 2004; Koizumi et al., 2009; Onan et al., 2009; Gu et al., 2015; Amarasekara et al., 2018). Additionally, we detected a significantly higher expression of VCAM-1 by dexa-treated JPCs. VCAM-1 was shown to mediate local recruitment of osteoclast progenitors and to promote osteoclastogenesis (Lu et al., 2011; Hayashi et al., 2013).

In this study, we show only certain, but not all, effects of dexa on JPC behavior. Considering the high clinical relevance of GCs and their negative effects on bone stability, the underlying mechanisms should be investigated in-depth in the future. To fully understand these mechanisms transcriptome and proteome analyses would be necessary.

The methods used in our study can also serve as a model for studying bone formation, fracture healing, and various pathological conditions such as (glucocorticoid-induced) osteoporosis, osteoarthritis, bone cancer, and others, in which the interactions of osteoblasts with surrounding cells play a key role.

## 5 Conclusion

In the present study, we report on multiple effects of the glucocorticoid dexamethasone (dexa) on the mineralization, extracellular matrix (ECM) composition, gene expression, and paracrine secretion of jaw periosteum-derived MSCs (JPCs).

The influence of dexa on the phenotype and behavior of cells during osteogenic differentiation has to be considered when evaluating the results of *in vitro* studies. Since we demonstrated in the present study that omission of dexa has positive effects on osteogenic differentiation while reducing osteoclast activation, standard dexa supplementation of the osteogenic medium must be critically evaluated.

We hypothesize that the osteoclastogenic effect of JPCs treated with dexa highlights a possible role of periosteal cells in the pathology of glucocorticoid-induced osteoporosis (GIOP).

## Data Availability

The original contributions presented in the study are included in the article/Supplementary Material; further inquiries can be directed to the corresponding authors.
