# Supplementary figures and images for: How osteogenic is dexamethasone?—effect of the corticosteroid on the osteogenesis, extracellular matrix, and secretion of osteoclastogenic factors of jaw periosteum-derived mesenchymal stem/stromal cells
Source: Front Cell Dev Biol. 2022 Oct 31;10:953516. doi: 10.3389/fcell.2022.953516 (PMC9660266; doi:10.3389/fcell.2022.953516)

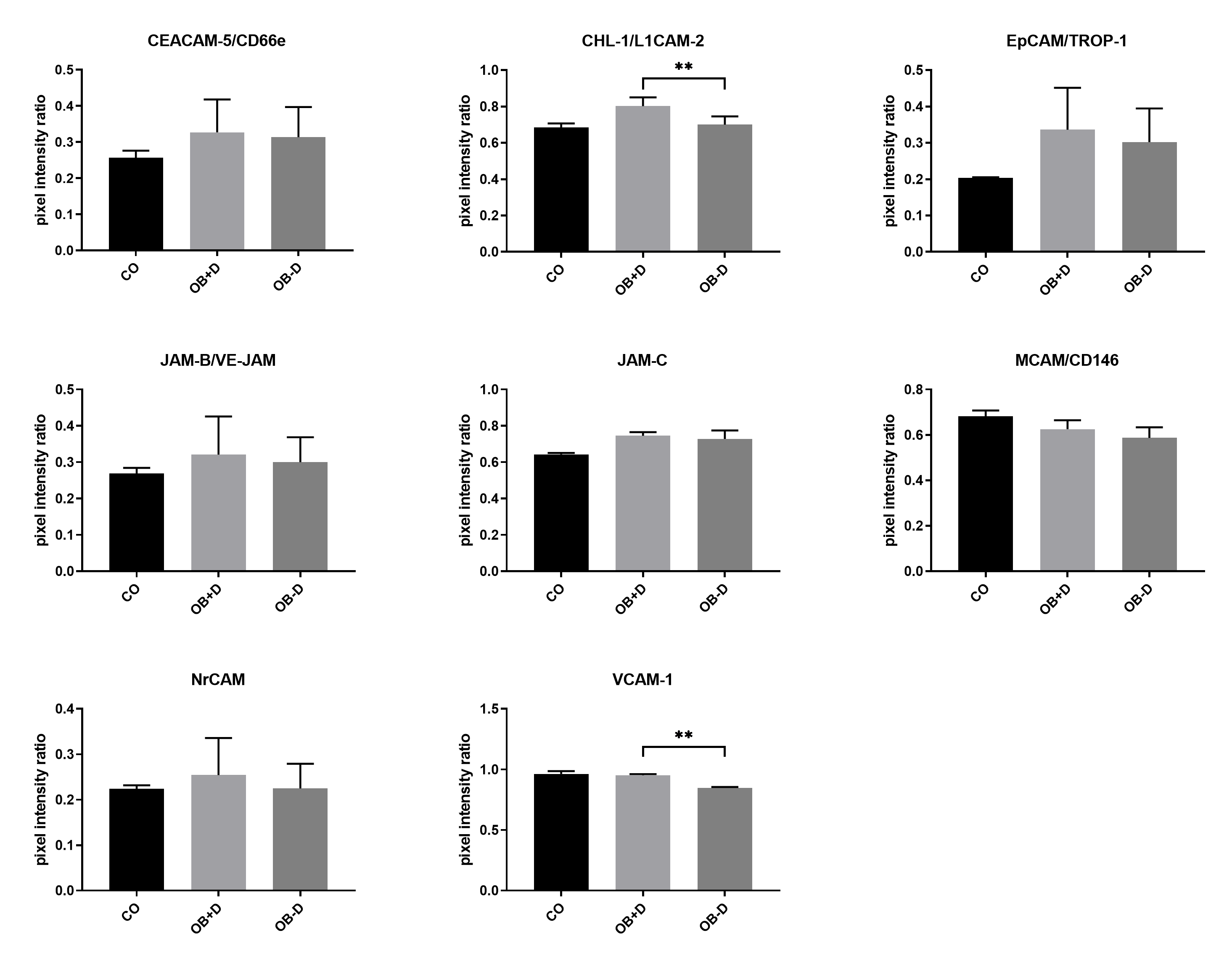

Supplement: Supplementary file 1 [file Image6.TIF]

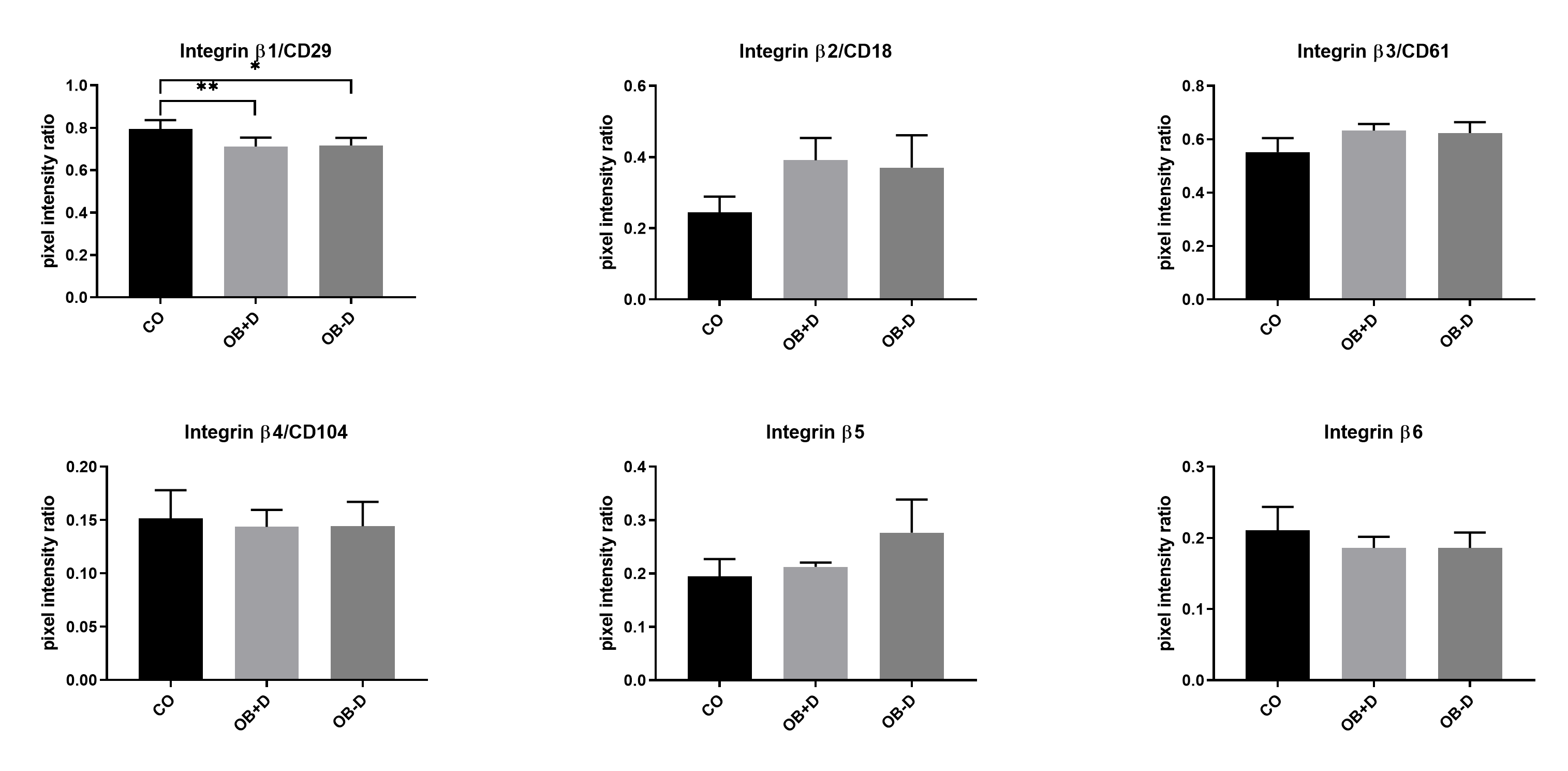

Supplement: Supplementary file 3 [file Image3.TIF]

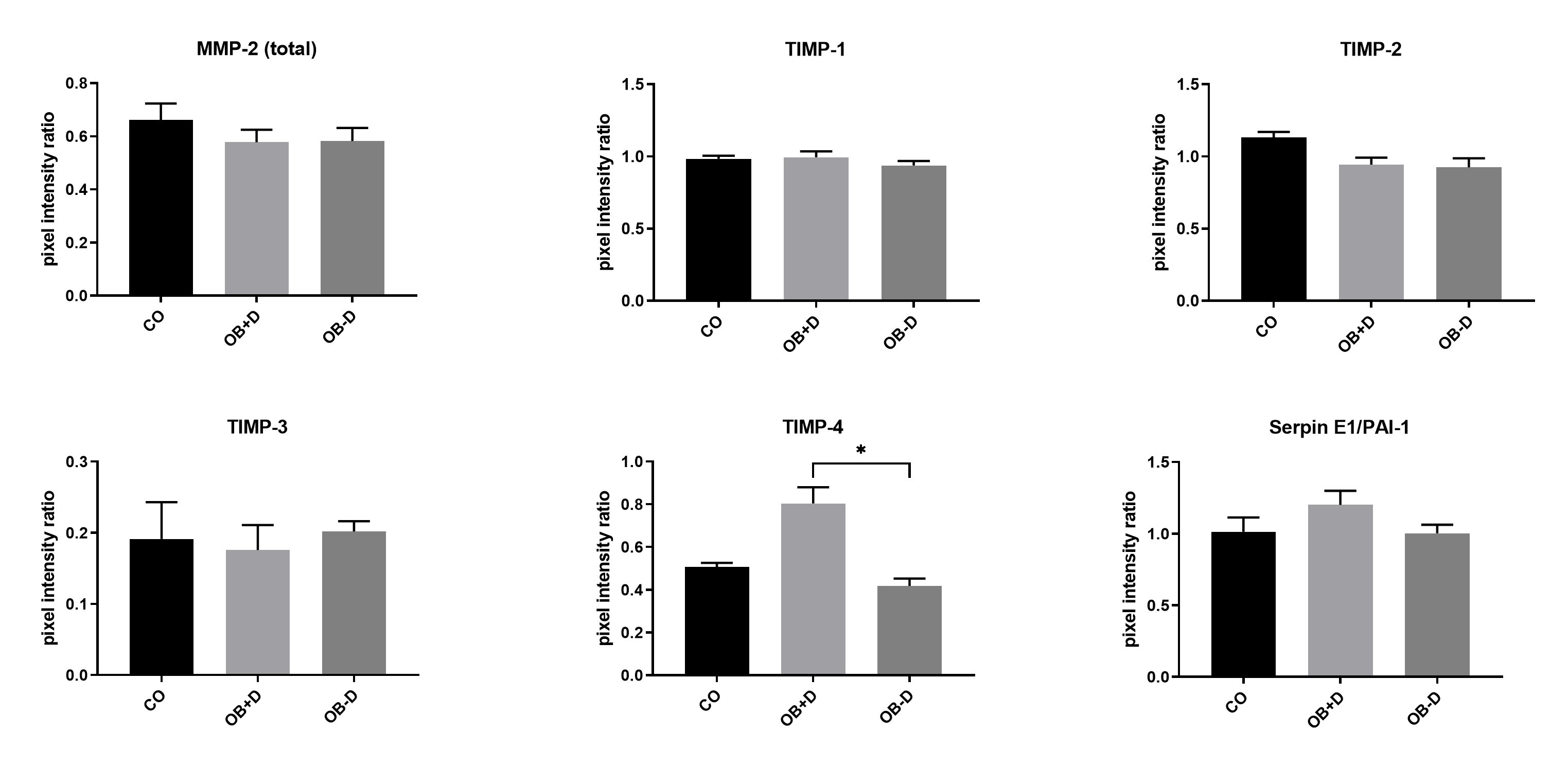

Supplement: Supplementary file 4 [file Image4.TIF]

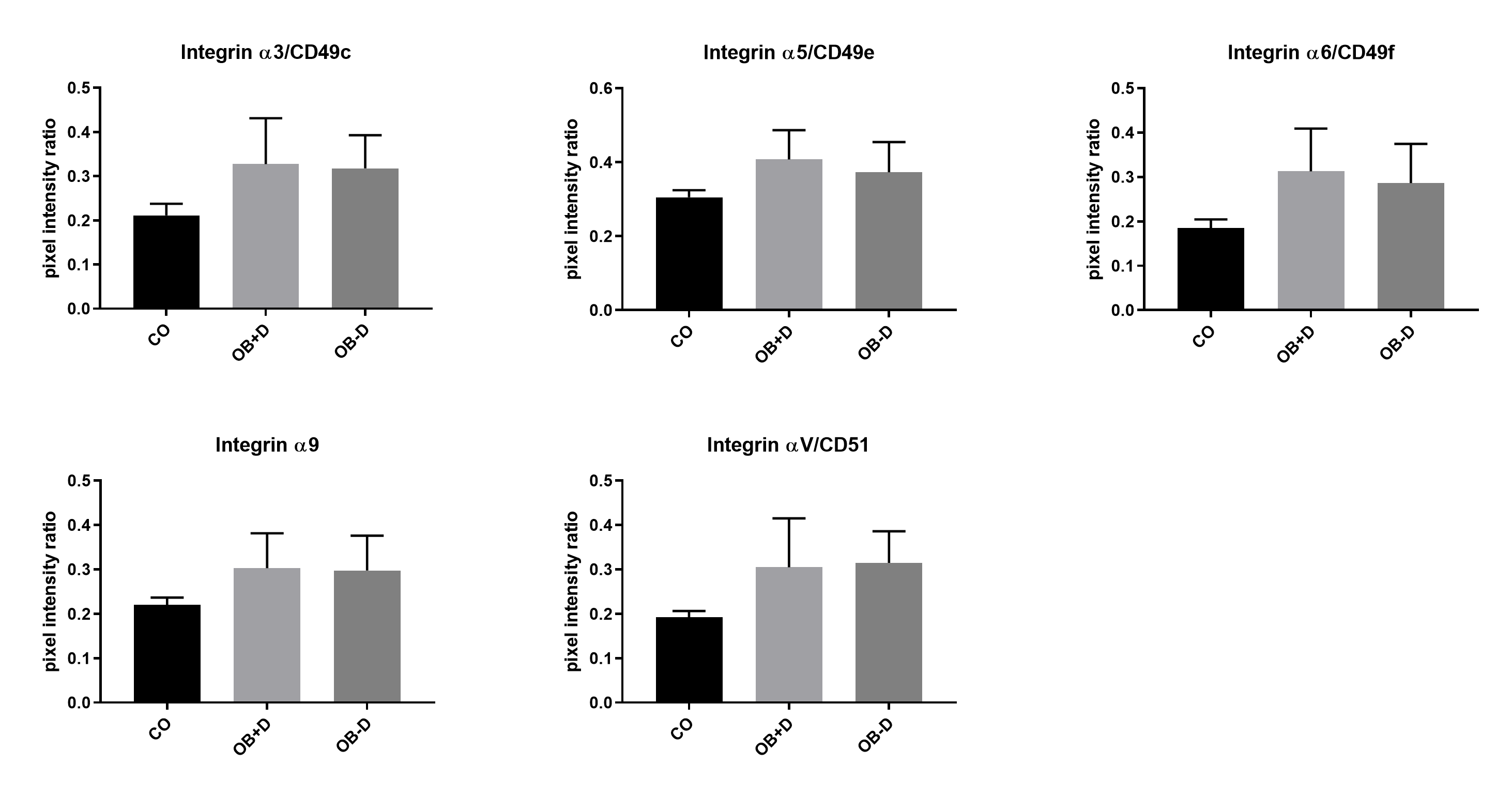

Supplement: Supplementary file 5 [file Image2.TIF]

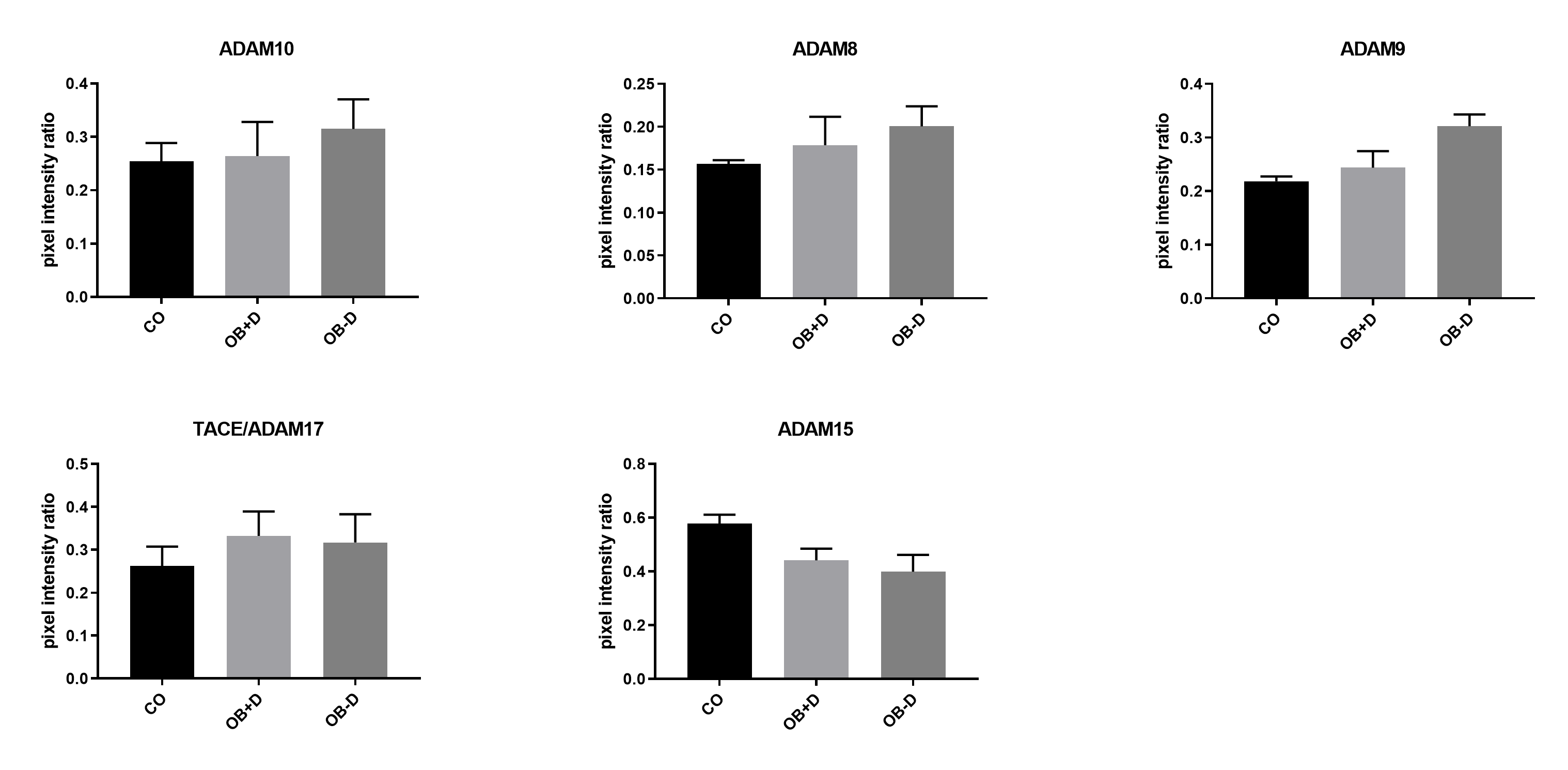

Supplement: Supplementary file 6 [file Image1.TIF]

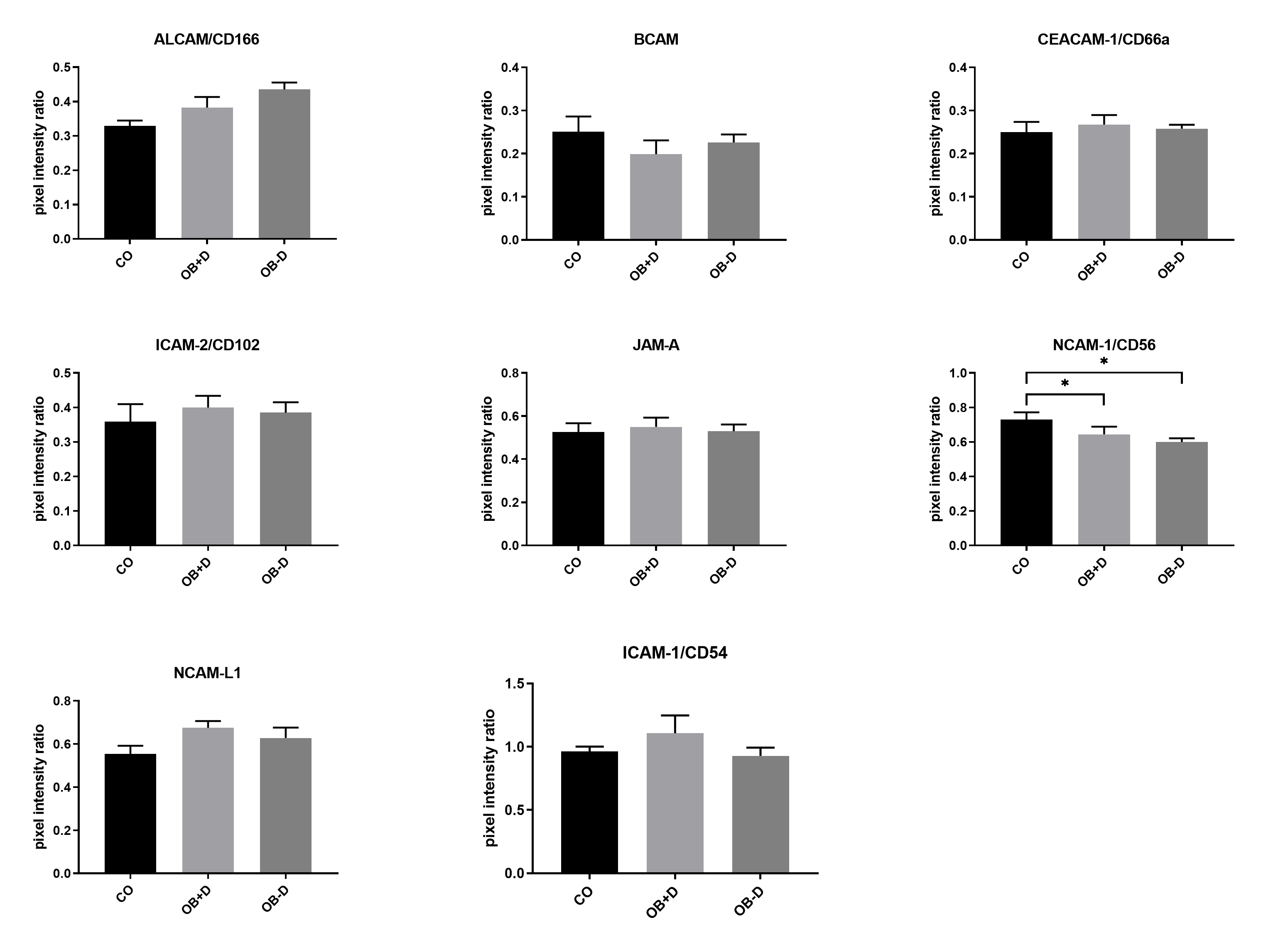

Supplement: Supplementary file 7 [file Image5.TIF]
