# Supplementary material for: How osteogenic is dexamethasone?—effect of the corticosteroid on the osteogenesis, extracellular matrix, and secretion of osteoclastogenic factors of jaw periosteum-derived mesenchymal stem/stromal cells
Source: Front Cell Dev Biol. 2022 Oct 31;10:953516. doi: 10.3389/fcell.2022.953516 (PMC9660266; doi:10.3389/fcell.2022.953516)
Supplement: Supplementary file 2 [file Table1.DOCX]

Supplementary Material

## Protein array data

### ADAMs

Figure 1: Secretion of A Disintegrin And Metalloproteinases (ADAMs) by JPCs incubated for 15 days with control medium (CO) and osteogenic medium with (OB+D) and without (OB-D) dexa. Protein expression was examined by detection of soluble factors secreted into culture supernatants proteome profiler arrays and analysis of pixel intensities using ImageJ software. Pixel intensity ratio mean ± SEM values were calculated and compared using one-way ANOVA and Tuckey’s multiple comparison test (n=3).

### α-Integrins:

Figure 2: Secretion of α-Integrins by JPCs incubated for 15 days with control medium (CO) and osteogenic medium with (OB+D) and without (OB-D) dexa. Protein expression was examined by detection of soluble factors secreted into culture supernatants proteome profiler arrays and analysis of pixel intensities using ImageJ software. Pixel intensity ratio mean ± SEM values were calculated and compared using one-way ANOVA and Tuckey’s multiple comparison test (n=3).

### β-Integrins:

Figure 3: Secretion of β-Integrins by JPCs incubated for 15 days with control medium (CO) and osteogenic medium with (OB+D) and without (OB-D) dexa. Protein expression was examined by detection of soluble factors secreted into culture supernatants proteome profiler arrays and analysis of pixel intensities using ImageJ software. Pixel intensity ratio mean ± SEM values were calculated and compared using one-way ANOVA and Tuckey’s multiple comparison test (n=3, *=p<0.05, **=p<0.01).

### TIMPs, MMP2, Serpin:

Figure 4: Secretion of MMP-2, TIMPs and Serpin by JPCs incubated for 15 days with control medium (CO) and osteogenic medium with (OB+D) and without (OB-D) dexa. Protein expression was examined by detection of soluble factors secreted into culture supernatants proteome profiler arrays and analysis of pixel intensities using ImageJ software. Pixel intensity ratio mean ± SEM values were calculated and compared using one-way ANOVA and Tuckey’s multiple comparison test (n=3, *=p<0.05).

### CAMs:

Figure 5: Secretion of cell adhesion molecules (CAMs) by JPCs incubated for 15 days with control medium (CO) and osteogenic medium with (OB+D) and without (OB-D) dexa. Protein expression was examined by detection of soluble factors secreted into culture supernatants proteome profiler arrays and analysis of pixel intensities using ImageJ software. Pixel intensity ratio mean ± SEM values were calculated and compared using one-way ANOVA and Tuckey’s multiple comparison test (n=3, *=p<0.05, **=p<0.01).

### Cadherins:

Figure 6: Secretion of cadherins by JPCs incubated for 15 days with control medium (CO) and osteogenic medium with (OB+D) and without (OB-D) dexa. Protein expression was examined by detection of soluble factors secreted into culture supernatants proteome profiler arrays and analysis of pixel intensities using ImageJ software. Pixel intensity ratio mean ± SEM values were calculated and compared using one-way ANOVA and Tuckey’s multiple comparison test (n=3, *=p<0.05).

## Supplementary Tables

Supplemental Table 1: Gene expression of osteogenic markers by JPCs cultured with control medium (CO) and osteogenic medium with (OB+D) and without dexa (OB-D). Mean ± SEM values of mRNA copy numbers relative to the housekeeping gene GAPDH (n=7) are listed.

|  | **Gene expression ratio mean ± SEM [1/GAPDH]** | | |
| --- | --- | --- | --- |
|  | **CO** | **OB+D** | **OB-D** |
| **ALP** | 2.42E-02 ± 6.30E-03 | 6.77E-02 ± 1.46E-02 | 9.61E-02 ± 1.34E-02 |
| **BSP2** | 6.28E-05 ± 2.00E-05 | 1.05E-04 ± 5.81E-05 | 3.08E-04 ± 1.56E-04 |
| **COL1A1** | 3.75E-01 ± 8.54E-02 | 1.46E-01 ± 3.05E-02 | 6.12E-01 ± 1.49E-01 |
| **OCN** | 3.47E-04 ± 9.49E-05 | 7.46E-04 ± 1.10E-04 | 8.71E-04 ± 1.59E-04 |
| **OGN** | 2.48E-02 ± 1.05E-02 | 7.18E-03 ± 1.83E-03 | 4.42E-02 ± 9.34E-03 |
| **OPG** | 1.37E-01 ± 4.78E-02 | 1.42E-02 ± 2.68E-03 | 4.83E-02 ± 1.44E-02 |
| **OPN** | 4.43E-05 ± 1.18E-05 | 1.03E-04 ± 3.42E-05 | 1.20E-04 ± 4.63E-05 |
| **OSN** | 6.44E-01 ± 1.60E-01 | 3.23E-01 ± 1.17E-01 | 6.78E-01 ± 2.37E-01 |
| **OSX** | 1.21E-05 ± 3.42E-06 | 4.37E-05 ± 1.80E-05 | 6.78E-05 ± 3.36E-05 |
| **POSTN** | 1.32E-02 ± 7.22E-03 | 2.09E-02 ± 9.75E-03 | 7.11E-02 ± 3.48E-02 |
| **RUNX2** | 1.17E-02 ± 2.70E-03 | 1.17E-02 ± 2.07E-03 | 1.67E-02 ± 2.67E-03 |

Supplemental Table 2: Expression of chondrogenic (COL2A1, COMP, SOX9) and adipogenic (LEP, LPL, PPARγ) marker genes by JPCs cultured with control medium (CO) and osteogenic medium with (OB+D) or without dexa (OB-D). Mean ± SEM values of mRNA copy numbers normalized to the housekeeping gene GAPDH (n=7).

|  | **Gene expression ratio mean ± SEM [1/GAPDH]** | | |
| --- | --- | --- | --- |
|  | **CO** | **OB+D** | **OB-D** |
| **COL2A1** | 4.90E-06 ± 1.30E-06 | 8.30E-06 ± 1.60E-06 | 1.30E-05 ± 3.40E-06 |
| **COMP** | 7.40E-04 ± 5.90E-04 | 2.60E-04 ± 1.10E-04 | 5.70E-04 ± 4.90E-04 |
| **LEP** | 9.10E-04 ± 2.40E-04 | 1.20E-02 ± 3.20E-03 | 3.20E-03 ± 1.60E-03 |
| **LPL** | 2.80E-05 ± 9.10E-06 | 7.40E-05 ± 2.00E-05 | 4.30E-05 ± 1.40E-05 |
| **PPARγ** | 1.10E-02 ± 3.20E-03 | 4.00E-02 ± 7.20E-03 | 3.10E-02 ± 7.00E-03 |
| **SOX9** | 1.60E-03 ± 2.90E-04 | 7.80E-04 ± 9.30E-05 | 2.80E-03 ± 4.80E-04 |

Supplemental Table 3: Expression of ECM related genes by JPCs cultured with control medium (CO) and osteogenic medium with (OB+D) or without dexa (OB-D). Mean ± SEM values of mRNA copy numbers relative to the housekeeping gene GAPDH are listed (n=7).

|  | **Gene expression ratio mean ± SEM [1/GAPDH]** | | |
| --- | --- | --- | --- |
|  | **CO** | **OB+D** | **OB-D** |
| **COL1A1** | 3.75E-01 ± 8.54E-02 | 1.46E-01 ± 3.05E-02 | 6.12E-01 ± 1.49E-01 |
| **COL1A2** | 2.26E+00 ± 2.72E-01 | 1.30E+00 ± 2.84E-01 | 3.56E+00 ± 9.96E-01 |
| **COL2A1** | 4.93E-06 ± 1.34E-06 | 8.27E-06 ± 1.58E-06 | 1.29E-05 ± 3.44E-06 |
| **COL7A1** | 1.15E-03 ± 2.51E-04 | 6.01E-03 ± 1.39E-03 | 1.60E-03 ± 3.88E-04 |
| **COL8A1** | 1.07E-02 ± 3.26E-03 | 3.13E-02 ± 9.48E-03 | 1.93E-02 ± 8.39E-03 |
| **COL10A1** | 5.81E-04 ± 2.42E-04 | 1.50E-03 ± 9.33E-04 | 1.55E-03 ± 5.07E-04 |
| **COL11A1** | 1.78E-02 ± 8.91E-03 | 3.19E-02 ± 1.28E-02 | 1.58E-02 ± 9.65E-03 |
| **COL12A1** | 5.38E-02 ± 1.40E-02 | 2.46E-02 ± 9.77E-03 | 2.43E-02 ± 5.42E-03 |
| **TIMP4** | 1.80E-03 ± 4.24E-04 | 2.31E-02 ± 3.44E-03 | 3.78E-03 ± 7.28E-04 |

Supplemental Table 4: Expression of osteoclastogenesis related genes by JPCs incubated with control medium (CO), osteogenic medium containing dexa (OB+D) and osteogenic medium without dexa (OB-D). Mean ± SEM values of mRNA copy numbers relative to the housekeeping gene GAPDH (n=7) are listed.

|  | **Gene expression ratio mean ± SEM [1/GAPDH]** | | |
| --- | --- | --- | --- |
|  | **CO** | **OB+D** | **OB-D** |
| **OPG** | 1.37E-01 ± 4.78E-02 | 1.42E-02 ± 2.68E-03 | 4.83E-02 ± 1.44E-02 |
| **RANKL** | 7.33E-04 ± 2.85E-04 | 1.03E-04 ± 2.71E-05 | 5.20E-04 ± 2.40E-04 |
| **IL-6** | 4.36E-02 ± 1.04E-02 | 1.23E-02 ± 4.44E-03 | 3.72E-02 ± 1.14E-02 |
| **IL-8** | 1.10E-03 ± 3.58E-04 | 1.47E-02 ± 3.90E-03 | 1.43E-03 ± 6.25E-04 |
| **IL-23** | 1.21E-06 ± 4.22E-07 | 3.41E-06 ± 7.89E-07 | 3.05E-06 ± 9.26E-07 |
| **IL-27** | 2.36E-05 ± 7.17E-06 | 3.27E-05 ± 6.36E-06 | 4.44E-05 ± 1.96E-05 |

**Table 5: Expression of osteoclast marker genes by PBMCs incubated with control medium (CO), osteogenic medium containing dexa (OB+D) and osteogenic medium without dexa (OB-D). Mean ± SEM values of gene expression induction (2^-ΔΔCt^) are listed.**

|  | **Relative gene expression mean ± SEM [2^-ΔΔCt^]** | | | |
| --- | --- | --- | --- | --- |
|  | **negative** | **positive** | **JPC_OB-D** | **JPC_OB+D** |
| **ITGB3** | 4.41E-01 ± 2.56E-02 | 1.18E+00 ± 3.91E-01 | 1.79E+00 ± 1.77E-01 | 5.12E+00 ± 3.56E-01 |
| **ACP5** | 9.56E-01 ± 2.67E-02 | 1.00E+00 ± 5.48E-02 | 5.42E-01 ± 1.84E-02 | 8.53E-01 ± 1.86E-02 |
| **CTSK** | 3.54E-01 ± 2.89E-02 | 1.13E+00 ± 3.27E-01 | 1.04E+00 ± 1.21E-01 | 3.22E+00 ± 1.13E-01 |
| **CALCR** | 3.77E-01 ± 9.14E-02 | 1.21E+00 ± 4.32E-01 | 1.00E+00 ± 1.14E-01 | 5.20E+00 ± 5.94E-01 |
